# Amino acid compositions contribute to the proteins’ evolution under the influence of their abundances and genomic GC content

**DOI:** 10.1038/s41598-018-25364-1

**Published:** 2018-05-09

**Authors:** Meng-Ze Du, Shuo Liu, Zhi Zeng, Labena Abraham Alemayehu, Wen Wei, Feng-Biao Guo

**Affiliations:** 1 School of Life Science and Technology, University of Electronic Science and Technology of China, Chengdu, China; 2 School of Life Sciences, Chongqing University, Chongqing, China; 3 Centre for Informational Biology, University of Electronic Science and Technology of China, Chengdu, China; 4 Key Laboratory for Neuroinformation of the Ministry of Education, University of Electronic Science and Technology of China, Chengdu, China

## Abstract

Inconsistent results on the association between evolutionary rates and amino acid composition of proteins have been reported in eukaryotes. However, there are few studies of how amino acid composition can influence evolutionary rates in bacteria. Thus, we constructed linear regression models between composition frequencies of amino acids and evolutionary rates for bacteria. Compositions of all amino acids can on average explain 21.5% of the variation in evolutionary rates among 273 investigated bacterial organisms. In five model organisms, amino acid composition contributes more to variation in evolutionary rates than protein abundance, and frequency of optimal codons. The contribution of individual amino acid composition to evolutionary rate varies among organisms. The closer the GC-content of genome to its maximum or minimum, the better the correlation between the amino acid content and the evolutionary rate of proteins would appear in that genome. The types of amino acids that significantly contribute to evolutionary rates can be grouped into GC-rich and AT-rich amino acids. Besides, the amino acid with high composition also contributes more to evolutionary rates than amino acid with low composition in proteome. In summary, amino acid composition significantly contributes to the rate of evolution in bacterial organisms and this in turn is impacted by GC-content.

## Introduction

The rate and mechanism of protein sequence evolution have been central questions in evolutionary biology since the 1960s^1^. The rate of protein evolution is generally thought to reflect the relative importance of selection and genetic drift, and is used to identify selective forces acting on genomes^2^. Protein sequence evolution has been investigated at the level of the DNA codons by examining amino acids and codons^3^, Rates of protein evolution are usually estimated by calculating the number of amino acid substitutions per site between a pair of orthologous proteins^1^. The ratio of the number of nonsynonymous nucleotide substitutions per nonsynonymous site (*Ka)* to the number of synonymous nucleotide substitutions per synonymous site (*Ks*) is frequently computed to assay the strength and direction of selection^4–6^. Theoretical and empirical analyses have demonstrated the importance of selection against errors in molecular and cellular processes, i.e., purifying selection in protein evolution. Determining the selection pressures of protein evolution that have shaped genetic variation forms a major part of many studies of molecular evolution.

Amino acids tend to be gained and lost with a universal trend during protein evolution^7^, and the biased amino acid usage is thought to be related to the molecular weights, protein structure, and the cost selection for synthesis^8–10^. However, our knowledge on the relationship of amino acid composition and the substitution rate are not consistent. Graur reported a highly significant correlation between the nonsynonymous substitution rate (*Ka*) and amino acid composition in mammalian proteins (*R*^2^: ~0.38), and thus he proposed that composition and changeability of amino acids are the main factor determining evolutionary rate rather than other factors such as functionality^11^. Conversely, another work concluded that rates of protein evolution was only weakly affected by amino acid compositions (*R*^2^: <0.10)^12^. By constructing an integrated probabilistic modeling approach in *Saccharomyces cerevisiae*, it was observed that amino acid composition together with protein abundance strongly contribute to the models of predicting slowly evolving proteins^13^. Recent research investigated the evolutionary patterns of amino acids in eight primates and concluded that amino acid usage was an important factor for protein evolution^14^. Although these findings hint at a close relationship between amino acid composition and evolution, direct computational and experimental results have been rare for bacterial organisms.

Thus, we systematically investigated the relationship between amino acid composition and evolutionary rates in 273 bacteria belonging to 18 phyla. The evolutionary rate *Ka/Ks*, which reflects the type and extent of selection pressure acting on genes^15^, was supposed to correlate with the compositions of 20 amino acids. In this work, we constructed multiple linear regression models (MLR) between evolutionary rates and amino acid composition for these bacterial species using ridge regression. Moreover, significant linear models were adopted to further analyze the contribution of each amino acid to evolutionary rate. Comparing individual contributions of the 20 amino acids, we found that the genomic GC content and amino acid richness had effects on the contribution of amino acid compositions to the evolutionary rates. This work confirmed the correlation between amino acid composition and *Ka/Ks* in bacteria and revealed that GC content and richness of amino acids influence their contributions to evolutionary rates.

## Materials and Methods

### Related species

**Figure 1:**
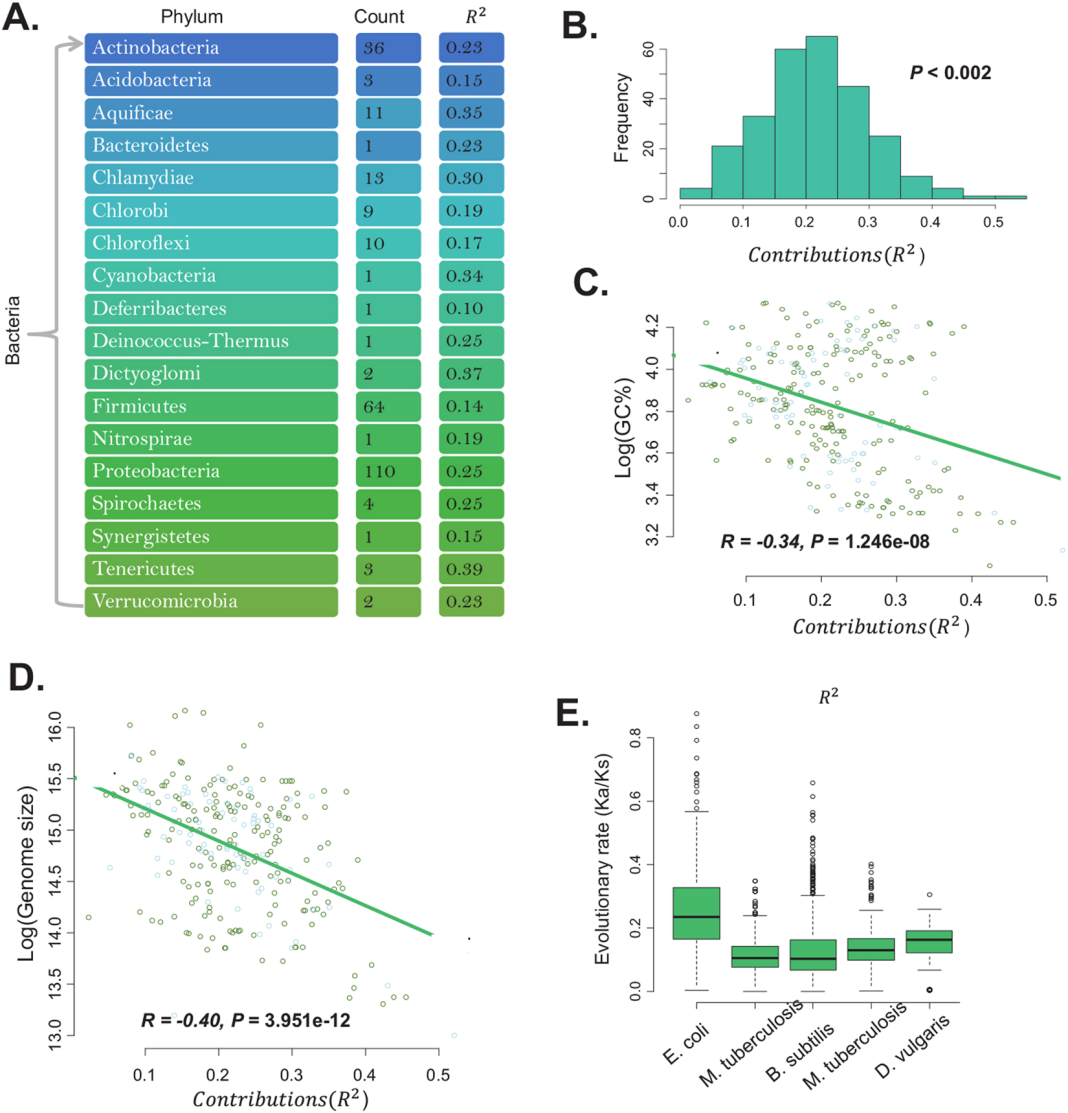
Multivariate linear regression models between amino acids and evolutionary rates. (**A**) There are 273 genome pairs belong to 18 phyla. Corresponding genome pair count and the average R^2^ for the multivariate linear regression between amino acid compositions and evolutionary rates were shown. (**B**) For 273 organisms, the total decision coefficient R^2^ ranged in 0~0.6 with P is less than 0.05. (**C**) GC content influences the total decision coefficient R^2^ for the multivariate linear regression between amino acid compositions and evolutionary rates. (**D**) Genome size negatively correlates with the total decision coefficient R^2^. (**E**) The evolutionary rates for proteins in the five model organisms and corresponding average are: 0.26, 0.11,0.13,0.16, and 0.15.

There are 273 genome pairs, belonging to 18 phyla (Fig. 1A), used to calculate the evolutionary rates of proteins (Table S1). For organisms with multiple chromosomes, the maximal chromosome was used. The following steps were applied to determine the reference genome. We downloaded all bacterial genomes in year 2017 from Genbank, which is a comprehensive public database for nucleotide sequences and biological annotation^16^. Organisms can be clustered into species, genuses, families, then orders, then classes, then phyla, and finally into kingdoms. We employed the taxonomy of bacteria to determine the best reference organisms: for genomes belonging to the same genus (if none, then the same family/order), we choose the genome having similar genome size as the reference of the target genomes. For example, the reference of NC_000117 (*Chlamydia trachomatis* D/UW-3/CX) is NC_015408 which belongs to the same genus *Chlamydia* containing more than 50 organisms.

### Evolutionary rates

Orthologous gene pairs between each genome pair were identified based on reciprocal best hits using the protein-protein BLAST service Blastp (https://blast.ncbi.nlm.nih.gov/Blast.cgi)^17,18^ with criteria of *E* <10^−5^, 80% minimum residues that could be aligned, and 30% identity. Protein sequences encoded by identified orthologous gene pairs were aligned with ClustalW^19^, and then back-translated into nucleotide sequences based on their original sequences. Numbers of substitutions per nonsynonymous site (*Ka*) and numbers of substitutions per synonymous site (*Ks*) were calculated following Yang’s definition using the PAML package^20,21^ with default parameters.

### Frequency of optimal codons

Codon usage bias for each gene was measured by the frequency of optimal codons (Fop). The optimal codons were described as that the most frequently used ones in a set of highly expressed genes for a certain species^22–24^. The ribosome proteins were used as the referenced highly expressed proteins. For a total of 273 organisms, Fops ranged from 0.36 (genes with the same codon bias) to 1.0 (genes with a strong codon preference).

### Protein abundance

The data of protein abundance was acquired from Paxdb (http://pax-db.org) which reprocessed, unified, quality-scored, and then integrated those qualification data^25^. The integrated data for five model organisms were downloaded: *Bacillus subtilis* subsp. subtilis str. 168 (*B. subtilis*), *Desulfovibrio vulgaris* str. Hildenborough (*D. vulgaris*), *Escherichia coli* str. K-12 substr. MG1655 (*E. coli*), *Mycobacterium tuberculosis* H37Rv (*M. tuberculosis*), *Neisseria meningitidis* MC58 (*N. meningitidis*), and *Streptococcus pyogenes* M1 GAS (*S. pyogenes*).

### Statistic methods

To avoid multi-collinearity, we adopted the ridge regression package in R language to choose variables^26,27^. Then the multivariate linear regression models were constructed using the chosen variables and evolutionary rates. Finally, we acquired 273 linear models with significant *P* values (<0.05). Furthermore, we also tried the principal component regression and the final total decision coefficients are close to the results of ridge regression we adopted in this paper, while the latter method can help us to refine to special amino acids.

## Results and Discussion

### Evolutionary rates significantly correlate with amino acid compositions

Above mentioned inconsistent results have been reported on the correlation between the amino acid compositions and evolutionary rates in eukaryotes. In this work, we investigated the relationship between amino acid composition and evolutionary rate *Ka/Ks* for bacteria. The *Ka/Ks* can be viewed as the outcome variable in a regression setting, with the amino acid compositions as predictors. Then, our aim is to construct a model to predict the evolutionary rate using the amino acid compositions for proteins in an organism. However, the high-dimensional data and the complex relationships between amino acids could influence the steady state of the model. Ridge regression is a means of estimating regression coefficients when data are high-dimensional and/or contain correlated variables^27,28^, and it can be used to obtain stable parameter estimates through guiding the variable selection (to select those amino acid compositions which have significant effects on evolutionary rate).

These analyses were applied to 273 bacterial proteomes to construct linear models. All multivariate linear regression models (MLR) are significant with the average total decision coefficient (*R*^2^) being 0.215 (*P* < 0.05), which means the amino acid compositions can explain average 21.5% of evolutionary rates’ variation (Fig. 1B, Table S1). These results show that amino acid composition generally correlate with the evolutionary rate in bacteria.

For some organisms, the coefficients of determination (*R*^2^) are very low. For example, *Dehalococcoides* CBDB1 uid58413, *Escherichia coli* ED1a uid59379 and *Bordetella bronchiseptica* RB50, the corresponding *R*^2^ are 0.0238, 0.0420 and 0.0472. However, some organisms have higher coefficients of determination (*R*^2^), such as *Buchnera aphidicola* Tuc7, *Buchnera aphidicola* 5 A (Acyrthosiphon pisum), *Buchnera aphidicola* cinara tujafilinat, the *R*^2^ are 0.4384, 0.4553 and 0.5206. Comparing with the genomes with lower *R*^2^, the three genomes are small sized and AT-rich. Hence, genomic GC content and genome size may influence *R*^2^ (or contribution strength) of that genome. Further linear regression was performed between *R*^2^ and GC content/genome size, and the results showed that *R*^2^ correlate with GC content/genome size (Fig. 1C,D). And using different reference genome will acquire different value of *Ka*, *Ks* and *Ka/Ks*, and the *R*^2^ for the MLR may change. For example, we choose *Bacillus subtilis* spizizenii W23 uid51879 as the reference organism of *Escherichia coli* ED1a uid59379, the *R*^2^ is lifted from 0.0420 to 0.1671. The 10% genomes with the highest *R*^2^ own a slightly higher *Ka (0.2029 vs 0.2250), Ks (2.5407 vs 2.9360)* and *Ka/Ks (0.0996 vs 0.1016)* than that of the 10% genomes with the lowest *R*^2^ (Student’s t test: *P* < 2.2e-16). However, the difference of *R*^2^ for MLR caused by the choice of reference genome is unavoidable and under control. In this work, the impact was minimized by choosing the reference genomes based on the phylogenetic relationship of organisms, and restrictions on the average evolutionary rate *Ka/Ks* (<5).

Other factors may constrain the correlation between the amino acid compositions and evolutionary rate. Expression level has been identified as a leading determinant of the protein variation in the rate of sequence evolution among genes encoded in the same genome^29^. It is previously reported that expression level strongly predicts the evolutionary rates (*Ka*) for yeast proteins^30^. Additionally, a limited but statistically significant negative correlation between Fop and *Ka/Ks* was reported, which is indicative of a link between selection on protein sequence and selection on codon usage^31^. For five model organisms, two MLRs are constructed: one is for evolutionary rates and multi amino acid compositions, and the other is for the evolutionary rates and variables including amino acid compositions, Fop and abundance. Comparing with the first MLRs, the second MLRs have increased/decreased *R*^2^ for the five organisms (Table 1). The average evolutionary rates for proteins in these genomes ranged from 0.1~0.3 (Fig. 1E). The results showed that expression level and Fop tend to negatively correlate with evolutionary rate which is consistent with the results of previous researches^1^. The linear models showed that some amino acids contribute positively to the *Ka/Ks*, while other amino acids contribute negatively to the *Ka/Ks* (Table S1). Finally, our results showed that the amino acid compositions can predict more of the variance among evolutionary rates than does protein abundance and Fop.

**Table 1:** The linear models for evolutionary rates and abundance/Fop/amino acid compositions.

Organism	Reference genome	Homologous protein numbers	Linear regression models(Evolutionary ~)					
			abundance	Fop	amino acid compositions		abundance, Fop and amino acid compositions	
			${{\boldsymbol{R}}}^{{\bf{2}}}$	${{\boldsymbol{R}}}^{{\bf{2}}}$	${{\boldsymbol{R}}}^{{\bf{2}}}$	Variables significantly contribute to evolutionary rates(positive; negative)	${{\boldsymbol{R}}}^{{\bf{2}}}$	Variables significantly contribute to evolutionary rates(positive; negative)
E. coli (NC_000913)	NC_014479	610	0.0187	0.0536	0.1827	L,V,W; H,R,G,Y	0.1836	V,L,W; R,Q,G,H,Y,Fop
M. tuberculosis (NC_000962)	NC_015125	351	0.0368	0.2233	0.2968	A,V; I,D,K	0.3370	A,V; K,D,Fop
B. subtilis (NC_000964)	NC_014829	1134	0.0275	0.0899	0.2215	L,W,F,I,S,Y; D,N,P,E,R,G	0.2263	K,W,F,L,A,I,S; N,D,E,P,R,G,Fop
M. tuberculosis (NC_002737)	NC_007350	455	0.0349	0.0723	0.2582	V,Y,L,M; N,E,D,G,R,P	0.2422	V,Y,M; N,Fop,E,D,G,P,R
D. vulgaris (NC_002937)	NC_006832	127	0.0101#	0.0748	0.2547	V;N,H,Y,I	0.2332	V; N,Y,I,H

^*^All MLRs in this table have *P* values less than 0.05. ^#^This linear model has *P* = 0.45, which is nonsignificant.

Further comparative analyses were performed to check whether organisms with close evolutionary relationship have similar tendency of amino acid contributions to evolutionary rates. To acquire the predictive power of constructed regression models, we compared the linear models for organisms from one genus. The model organism *Escherichia coli* have 29 different strains can be downloaded from Genbank^16^. When take NC_011740 as reference genome, we found that amino acid Cys (C) and Ser (S) positively contribute to evolutionary rates, while Gly (G) negatively contribute to evolutionary rates in the built linear models for all these 29 strains. When take NC_014479 as reference genome, we found that amino acid Leu (L) and Val (V) positively contribute to evolutionary rates, while Tyr (Y) negatively contribute to evolutionary rates in the built linear models for all these 29 strains (Table S2). The other genomes which belong to one genus also show similar tendency of amino acid contributions to evolutionary rates (Table S1). To some degree, we can predict the contribution of each amino acid to the evolutionary rate for closely related species with the constructed regression models.

### GC content influences the contribution of amino acids to evolutionary rates

The base composition of genomic sequences varies widely. GC-content, determining the underlying causes (selective or neutral) of base variations, is a major issue in genetics^32,33^. Bohlin *et al*. reported that amino acid usage is strongly linked with genomic AT content^34^ and Zhou *et al*. demonstrated that base usage, codon usage patterns and amino acid usage change with GC content with a linear correlation in prokaryotic organisms^35^. Thus, GC content plays a role both in the evolution of proteins and shaping amino acid composition. The abovementioned result showed that the *R*^2^ varied among different genomes and could the GC content be one of the controlling factors?

**Figure 2:**
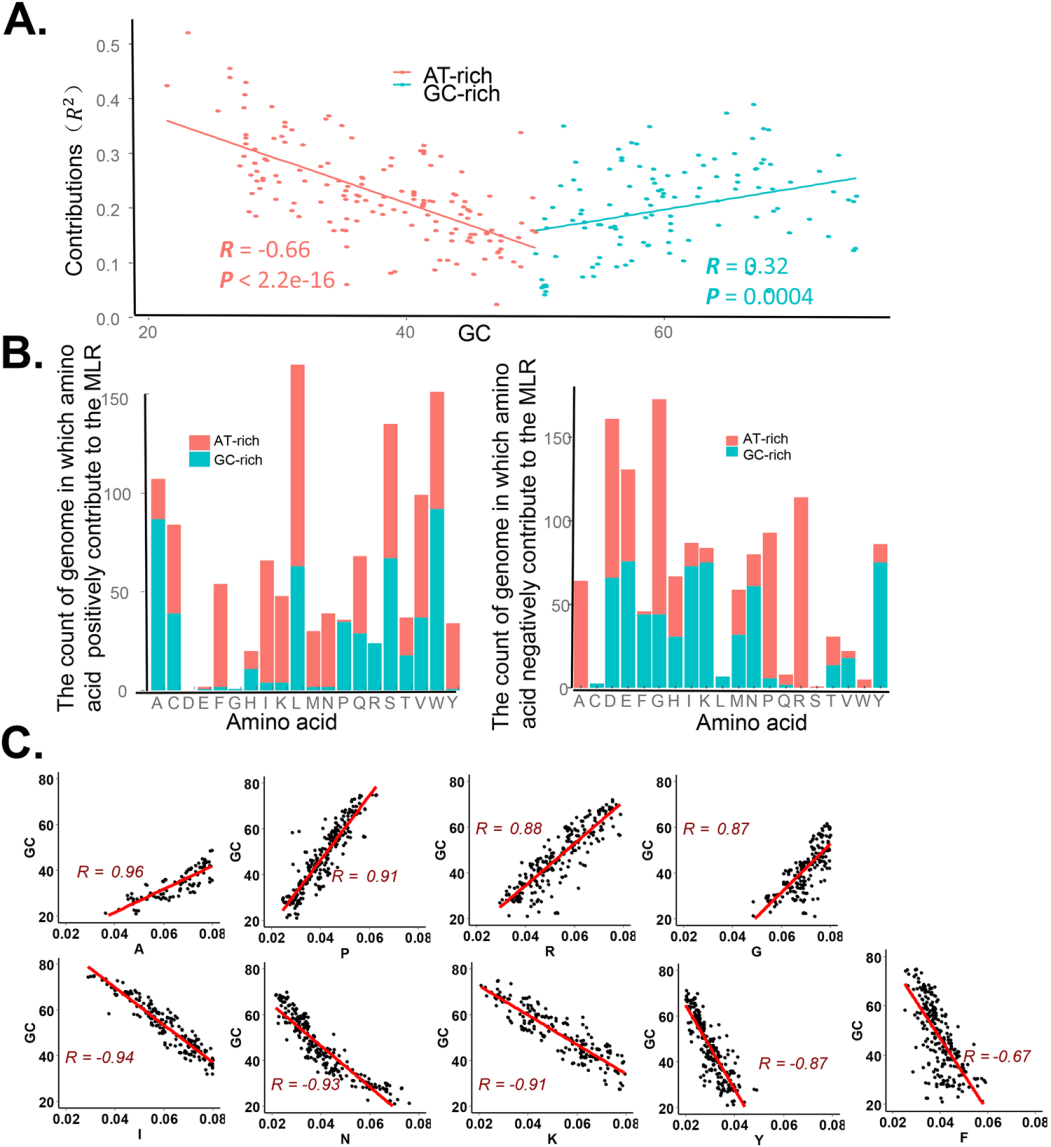
GC content influences the contributions of amino acid compositions to the evolutionary rates in GC-rich organisms and AT-rich organisms. (**A**) The relationship between GC content and the contributions of amino acid compositions to evolutionary rates for MLRs. (**B**) The count of the genomes that amino acid compositions negatively/positively contribute to the MLR in GC-rich and AT-rich organisms. The 20 amino acid types are represented by the letters A, C, D, E, F, G, H, I, K, L, M, N, P, Q, R, S, T, V, W and Y. (**C**) The point plots between amino acid compositions and GC contents. For GC-rich/AT-rich amino acids, the average compositions of these amino acids of 273 organisms are positively/negatively correlated with the GC content (*P* ≪ 0.05).

In this work, the coefficients of determination *R*^2^ for the MLR represent the intensity of the amino acid contribution to the evolutionary rates. Through the scatter plot where the x lab is GC content and the y lab is the coefficient of determination for MLR, we found two distinct patterns between GC-rich genomes and AT-rich genomes (Fig. 2A). For GC-rich genomes, the coefficient of determination grows along with the GC-content, while in AT-rich ones it grows with the AT-content. These results indicate that amino acids contribute more to evolutionary rates in organisms with either high GC content or high AT content.

The above results may be caused by biased compositions of amino acids encoded by codons with different GC contents. We thus extract those AAs which positively contribute to the evolutionary rate of the 273 linear models. The histograms of these amino acids show that GC-rich organisms and AT-rich organisms display different categories of AAs which contribute to evolutionary rates (Fig. 2B). The amino acids encoded by AT-rich codons (such as Phe, Ile, Tyr, Asn, Lys and so on) tend to positively contribute to MLRs for AT-rich organisms and negatively contribute to MLRs for GC-rich organisms. The amino acids encoded by GC-rich codons (Pro, Gly, Arg and Ala) tend to positively contribute to MLRs for GC-rich organisms and negatively contribute to MLRs for AT-rich organisms. The ratios of AT content to GC content for all codons encoding these amino acids are: Asn-5:1, Ile-5:1, Phe-5:1, Lys-5:1, Tyr-5:1, Ala-1:5, Pro-1:5, Arg-1:5, Gly-1:5. The results showed that in GC-rich organisms, amino acids owing more GC-rich codons contributed to the evolutionary rates more; while in AT-rich organisms, AAs owing more AT-rich codons contributed to the evolutionary rates more.

If the GC content largely determine the correlation between amino acids and evolutionary rates, the content of these amino acids among organisms should also change with the GC contents. Thus, the average amino acid compositions of the investigated 273 organisms were calculated. The four GC-rich amino acids Ala, Pro, Arg and Gly (A, P, R and G) positively correlate with GC contents of the organisms. The five AT-rich amino acids Phe, Ile, Tyr, Asn and Lys (F, I, Y, N and K) positively correlate with AT contents of the organisms (Fig. 2C). All these results supported that amino acid usage contribute to the evolutionary rates under the influence of GC-content.

### The content of special amino acid influences its contribution to evolutionary rates

**Figure 3:**
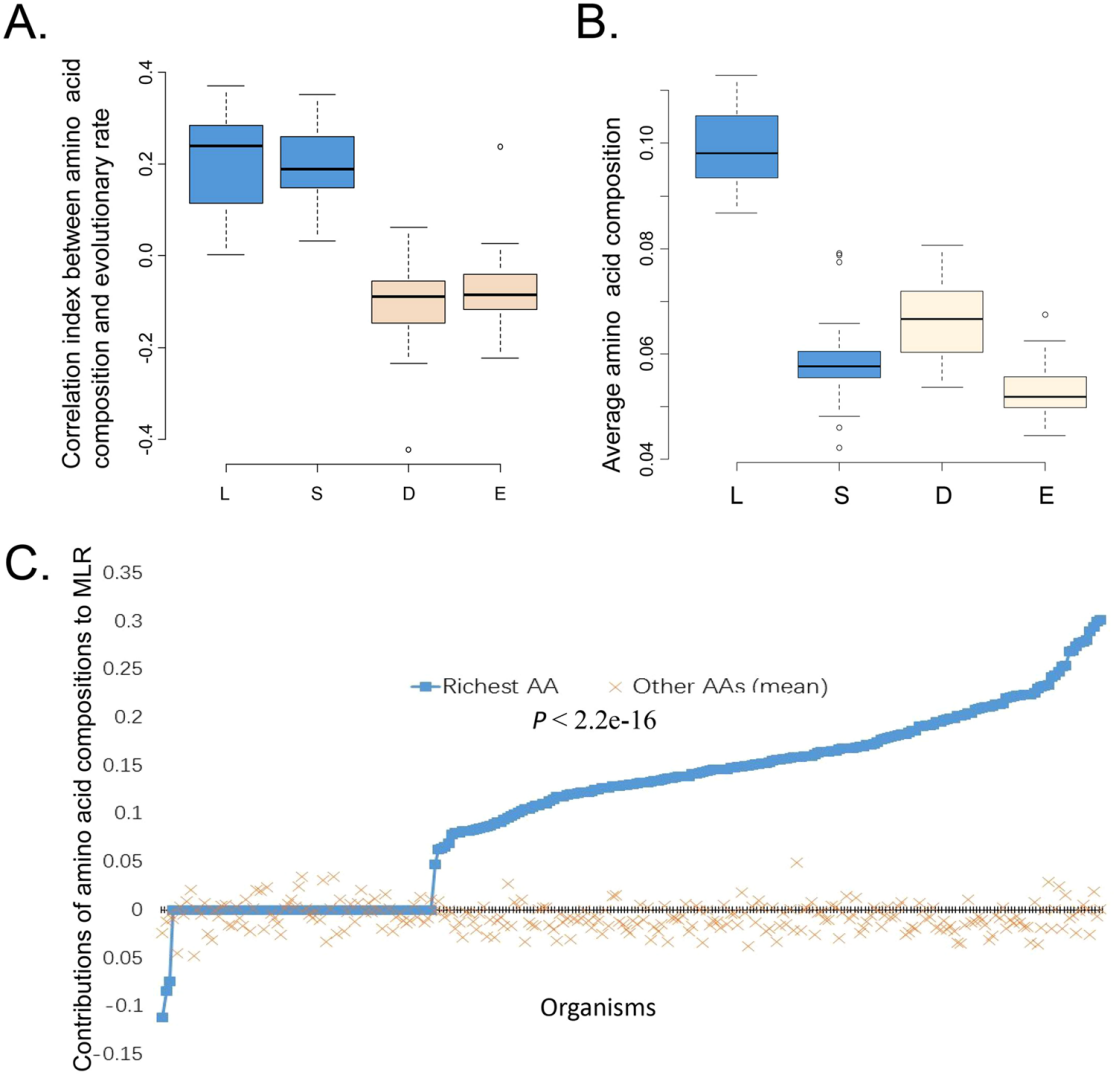
The amino acid composition and evolutionary rates. (**A**) The boxplot for the correlation index between amino acid composition and evolutionary rate in 56 genomes with GC content in range 45~55%. (**B**) The boxplot of the average amino acid compositions in 56 genomes with GC content in range 45~55%. (**C**) The scatterplot of contributions for 273 organisms. The horizontal axis represents organism. Each organism has two corresponding scatters: one is for the richest amino acids, and the other is for the rest amino acids.

From the above results, we observed that amino acid composition is partially influenced by GC content. But, the GC content is not the only factor influence amino acid compositions. We also observed that amino acids Asp and Glu (D and E) tend to be conservative in most organisms, while Leu and Ser (L and S) always contribute to the MLRs in most organisms. For organisms with GC content in range 45~55%, compositions of amino acid Leu and Ser positively correlate with the evolutionary rates, while the compositions of amino acids Asp and Glu negatively correlate with the evolutionary rates (Fig. 3A). In contrast, a significantly higher content of Leu than Asp and Glu were observed (Fig. 3B). Although the content of Ser is not higher than the content of Asp and Glu, the Ser is the precursor of amino acids Gly and Cys, according to the amino acid biosynthetic pathways^9^, which may result in that higher correlation between compositions of Ser and the evolutionary rates. If richer amino acids tend to contribute more to evolutionary rates than the rest, then these results can be explained.

To further prove that the richness of amino acid influences their contribution to evolutionary rates, we investigated the contributions of richest amino acids. Among the 273 linear models, the richest amino acids positively contribute in 71% of inspected organisms, and the rarest amino acids only positively contribute in 34% of those organisms. In 65 linear models where the richest and rarest amino acids both significantly contribute, the contributions of richest amino acids are lower than that of the rarest amino acids only in 9 organisms. Additionally, the contributions of the richest amino acids in 273 organisms not only higher than the rarest amino acids (Students’ t test: *P* < 2.2e-16), they also are higher than the average of the rest 19 types of amino acids (Students’ t test: *P* < 2.2e-16). For these organisms, the richest amino acids for the 52%, 30%, 14% and 4% genomes are Leu, Ala, Ile and Lys. This help to explain why in most organisms the compositions of amino acid Leu positively contribute to the evolutionary rates. Next, comparison of the contributions to evolutionary rates by the richest AAs and the rest 19 AAs show that richest AAs absolutely contribute more than the rest (Fig. 3C).

Our result showed that the higher the usage of special amino acid in proteome, the higher contributions the amino acid has to the evolutionary rate for this organism. The metabolic efficiency may result in the variation of amino acid composition. However, the statistical aberrations also cannot be ignored through magnifying the contribution of richest amino acids. This finding can be applied to understand, predict and even affect the evolution of leucine-rich genes^36^, alanine-rich genes^37^ and others.

## Discussion

Understanding the sequence and structure of proteins is important in understanding genome evolution. We looked into the relation between amino acid compositions and evolutionary rates in multiple organisms. Our results supported the conclusion that amino acid composition generally correlates with the evolutionary rates in bacterial organisms. Through ridge regression and multivariate linear regression, we acquired the contributions of different amino acids. Connecting these determination coefficients of MLR with the GC content and the rank of amino acid composition, we observed a trend for the co-variation of amino acid composition and evolutionary rate.

**Figure 4:**
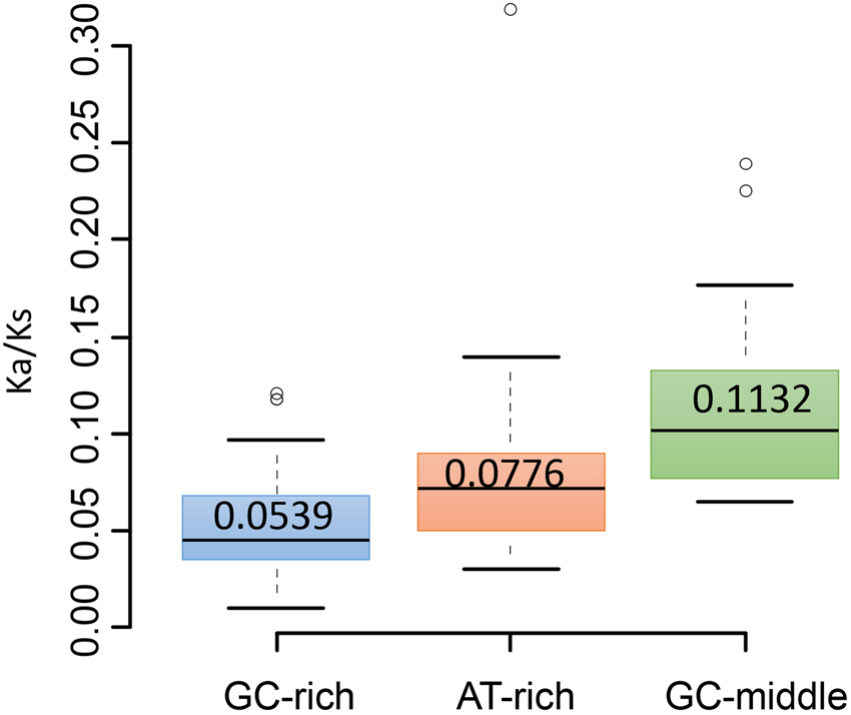
Average Ka/Ks for groups of genomes with different GC contents. The boxplot of Ka/Ks for GC-rich organism (GC content > 64%), AT-rich genomes (GC < 32%) and GC-middle genomes (GC content: 47% ~53%). The mean Ka/Ks for the three groups are: 0.0539, 0.0776 and 0.1132. The student’s t test showed that the GC-rich group and AT-rich group are significantly lower than the GC-middle group (*P* = 4.111e-07). The increase in Ka/Ks ratio may be an evidence of the relaxation of negative selection.

The result showed that the closer the GC-content of one genome to its maximum or its minimum, the better the correlation between the amino acid content and the evolutionary rate of proteins for that genome. Why evolutionary rates in genomes with extreme GC content have better correlation with amino acid compositions? In this paper we measured the evolutionary rate through the ratio between Ka and Ks for genes. Any increase/decrease of each of the two indexes can cause the ratio change. A prominent result showed that for GC-rich genomes, the usage of GC-rich amino acids (such as Ala, Pro, Arg and Trp) increase (Fig. 2C). However, the increase in the usage of these GC-rich amino acids under the influence of GC pressure may be limited by negative selection. If it is allowed, all other amino acids are turning to GC-rich amino acids (such as Ala, Pro, Arg and Trp) throughout the whole sequence. The Ka/Ks also represent the selective pressure^38–40^, and thus we compared the selective pressure for genomes with different GC-content. Average Ka/Ks for each genome was computed, and GC-rich organism (GC content > 64%) and AT-rich genomes (GC < 32%) generally have lower Ka/Ks than Genomes with GC content between 47% and 53% (Fig. 4, students’ t test: *P* = 4.111e-07). The decrease of Ka/Ks ratio in GC-richest or AT-richest genomes possibly reflects a stronger negative selection for genes of them. But it was also caused by the decrease of mutation pressure for the whole genomes. This discovery can help us to understand how the GC content influences the amino acid composition during evolution.

Those richest amino acids generally positively contribute to evolutionary rates and their corresponding contributions always are higher than the others. We also found that Leu is so abundant in proteins, while Cys and Trp are so rare in proteins. There is no direct report to explain this observation. It can be understood from the view of energy efficiency. Chen *et al*. reported that efficiency trade-offs drive nucleotide usage in transcribed regions, which means cheaper nucleotides encode more expensive amino acids^41^. The synthesis cost of A + G > U + C and the Leu codons have more U + C. Thus, it has moderate nucleotide cost. In addition, Leu is one amino acid with moderate energy cost, while Trp is the most expensive amino acid^42^. The protein structure may also result in high abundance of Leu and low abundance of Trp. For example, the common leucine-rich repeat motif such as leucine zipper needs enough Leu^43^. Because of containing sulfur, the Cys composition in proteins is limited^44^. Furthermore, whether the algorithm for the alignment might contribute something into the obtained results that lower abundance amino acids have lower contributions to the evolutionary rates? Usually such rare amino acids as Cys and Trp have the higher score for the alignment than Leu. Probably, Cys and Trp rich proteins are not just evolve slower, but they are also aligned better^45^. More direct evidences are needed to explain the abundance of these amino acids and their evolution mechanism, which can finally help us understand the effects of amino acid composition to evolutionary rates.

## Electronic supplementary material

Supplementary Information
