# Supplementary material for: Amino acid compositions contribute to the proteins’ evolution under the influence of their abundances and genomic GC content
Source: Sci Rep. 2018 May 9;8:7382. doi: 10.1038/s41598-018-25364-1 (PMC5943316; doi:10.1038/s41598-018-25364-1)
Supplement: Supplementary file 1 — Supplementary Information [file 41598_2018_25364_MOESM1_ESM.pdf]

Amino acid compositions contribute to the proteins' evolution under the influence of their abundances and genomic GC content

Meng-Ze Du[1], Shuo Liu[1], Zhi Zeng[1], Labena Abraham Alemayehu[1], Wen Wei[2*], Feng-Biao Guo[1,3,4*]

1 School of Life Science and Technology, University of Electronic Science and Technology of China, Chengdu, China.

2 School of Life Sciences, Chongqing University, Chongqing, China.

3 Centre for Informational Biology, University of Electronic Science and Technology of China, Chengdu, China.

4 Key Laboratory for Neuroinformation of the Ministry of Education, University of Electronic Science and Technology of China, Chengdu, China

Supplementary Tables

Table S1 Total decision coefficients and amino acid contributions for the linear models between evolutionary rates and multi amino acid compositions

Table S2 Total decision coefficients and amino acid contributions for the linear models between evolutionary rates and multi amino acid compositions specially for E.coli strains

Table S1 Total decision coefficients and amino acid contributions for the linear models between evolutionary rates and multi amino acid compositions

Organism	Genome size (bp)	GC_content	Chromosome	Richest AA	Rarest AA	Orthologous Chromosome			R^2	Orthologous gene numbers	The contributions of 20 amino acids to the linear models																			
						Accession ID	Genome size (bp)	GC content			K	H	I	N	L	M	C	A	F	G	D	E	Y	R	S	P	Q	V	W	T
Chlamydia trachomatis D UW 3 CX uid57637	1042519	41.31	NC_000117	L	W	NC_015408	1106197	41.08	0.3049	593	-	-	-0.1438	-0.0719	0.1409	-	0.1792	-	-	-0.1773	-0.1467	-	-0.1086	-0.1225	0.2029	-	-	-	0.0784	-
Chlamydophila pneumoniae CWL029 uid57811	1230230	40.58	NC_000922	L	W	NC_010287	1038842	41.33	0.2295	614	-	-	-	-0.0778	0.2025	-0.1076	-	-	-	-0.1153	-0.1523	-	-	-0.1118	0.1775	-	0.0876	-	-	-
Bacillus subtilis 168 uid57675	4215606	43.51	NC_000964	L	C	NC_014829	4681672	36.52	0.2126	1155	-	-	0.0700	-0.1004	0.1127	-	-	-	0.0979	-0.1514	-0.0929	-0.1222	0.0707	-0.1420	0.0645	-0.0981	-	-	0.1088	-
Campylobacter jejuni NCTC 11168 uid57587	1641481	30.55	NC_002163	L	W	NC_009715	1971264	44.54	0.3208	736	0.1339	-0.0707	0.0961	0.1217	-	-	-	-	0.1339	-0.1345	-0.0853	-0.0953	-	-0.2221	-	-0.1388	-	-	-	-0.0764
Chlamydophila pneumoniae AR39 uid57809	1229853	40.57	NC_002179	L	W	NC_010287	1038842	41.33	0.2236	596	-	-	-	-0.0979	0.2213	-0.1108	-	-	-	-0.1041	-0.1466	-	-	-0.0872	0.1654	-	0.1087	-	-	-
Chlamydophila pneumoniae J138 uid57829	1226565	40.58	NC_002491	L	W	NC_010287	1038842	41.33	0.2276	612	-	-	-	-	0.2096	-0.1073	-	-	-	-0.1099	-0.1424	-	-	-0.1086	0.1809	-	0.0880	-	-	-
Bacillus halodurans C 125 uid57791	4202352	43.69	NC_002570	L	C	NC_014976	4093599	43.85	0.2263	1295	-0.1215	-	0.1074	-	0.1593	-	-	-	-	-0.0811	-0.1603	-	-	-0.0624	0.0791	-	0.0582	0.1124	0.0902	-
Caulobacter crescentus CB15 uid57891	4016947	67.21	NC_002696	A	C	NC_014100	4655622	67.67	0.1839	1800	-0.0603	-0.0491	-	-0.1038	0.0640	-	-0.0581	0.1305	-0.0686	-0.0709	-0.1253	-0.1219	-	0.0798	0.1028	0.1114	-	-	0.0809	-
Bordetella bronchiseptica RB50 uid57613	5339179	68.08	NC_002927	A	C	NC_002928	4773551	68.10	0.0472	1847	-	-	-	-	-	-0.0529	0.0553	0.0858	-0.0531	-	-	-	-	-	0.0466	0.0773	-	-0.0532	0.0480	-
Bordetella parapertussis 12822 uid57615	4773551	68.10	NC_002928	A	C	NC_010170	5287950	65.48	0.3436	2023	-0.1246	-	-0.1663	-0.1181	0.0594	-	-	0.1846	-0.0739	-	-0.0912	-0.1013	-0.0485	-	-	0.0793	-	-	0.0603	-0.0767
Bordetella pertussis Tohama I uid57617	4086189	67.72	NC_002929	A	C	NC_010645	3732255	61.58	0.2886	1669	-0.1138	-	-0.1564	-0.1110	-	-	-	0.2239	-0.1077	-	-0.1258	-0.0803	-	-	-	0.0514	-	-	-	-
Chlorobium tepidum TLS uid57897	2154946	56.53	NC_002932	L	W	NC_011027	2289249	55.80	0.1331	1278	-	-	-0.1296	-	0.1581	-	-	0.1102	-	-	-0.1040	-0.0874	-0.0598	-	0.0636	-	0.0565	-	0.0678	-0.0973
Corynebacterium diphtheriae NCTC 13129 uid57691	2488635	53.48	NC_002935	A	C	NC_004369	3147090	63.14	0.1896	649	-	0.1045	-	-0.1255	0.0970	-	-	0.1776	-	-0.1550	-	-0.1189	-0.1559	-	0.0974	-	-	-	0.1458	-
Dehalococcoides ethenogenes 195 uid57763	1469720	48.85	NC_002936	L	W	NC_013890	1360154	47.31	0.0928	836	-	-	-	-	0.1991	-	-	-0.0838	-	-0.0736	-0.0743	-	-	-0.0941	-	-	-	-0.1052	-	-
Corynebacterium glutamicum ATCC 13032 uid57905	3309401	53.81	NC_003450	A	C	NC_015859	3433007	67.15	0.1642	733	-0.1118	-	-	-	-	-0.0938	-	0.1311	-	-0.0828	-0.0949	-	-0.1904	-	0.0798	0.0944	-	-	0.1177	-
Colwellia psychrerythraea 34H uid57855	5373180	38.01	NC_003910	L	W	NC_009665	5229686	46.31	0.2053	1514	-	-0.0753	0.0791	-	0.1051	-	-	-	-	-0.1648	-0.1295	-0.1040	-	-0.1610	0.0643	-0.0662	0.0682	0.0919	0.0674	0.0772
Campylobacter jejuni RM1221 uid57899	1777831	30.31	NC_003912	L	W	NC_008599	1773615	33.31	0.3312	812	0.1307	-0.1311	-	-	-	-	-	-0.1087	-	-0.2341	-0.1236	-0.1196	-	-0.2111	-	-0.1537	-	-	-	-0.0887
Bacillus anthracis Ames uid57909	5227293	35.38	NC_003997	L	C	NC_010184	5262775	35.56	0.1292	3253	0.1043	-0.0419	0.0468	0.0818	-	0.0905	0.0531	-0.1449	-	-0.0940	-0.0894	-	0.0445	-0.0598	0.0550	-0.0810	-	-	0.0977	-
Buchnera aphidicola Sg Schizaphis graminum uid57913	641454	25.33	NC_004061	I	W	NC_014909	722593	27.51	0.3780	298	0.1394	-	0.2894	-	-	-	-	-0.1193	-	-0.2460	-0.1214	-0.1342	-	-0.1242	-	-	-	0.1214	-	-
Escherichia coli CFT073 uid57915	5231428	50.48	NC_004431	L	C	NC_011740	4588711	49.94	0.0548	2057	-	0.0660	-	-0.0450	-	-	0.0754	-	-0.0554	-0.0950	-	-0.0805	-	-	0.1188	-	-	-0.0761	-	-
Bradyrhizobium japonicum USDA 110 uid57599	9105828	64.06	NC_004463	A	C	NC_009485	8264687	64.92	0.2576	2977	-0.1528	-	-0.1500	-0.1219	-	-0.0501	-	0.1954	-0.0731	-	-0.0804	-0.1401	-	0.0897	0.0546	0.1484	-	-	0.0815	-
Chlamydophila abortus S26 3 uid57963	1144377	39.87	NC_004552	L	W	NC_003361	1173390	39.22	0.1921	722	-	-	-	-	-	-	0.1917	-0.1013	-0.0839	-0.1610	-0.0819	-0.1321	-	-	0.1993	-	0.1252	0.1285	-	0.0884
Anaplasma marginale Maries uid57629	1197687	49.76	NC_004842	L	W	NC_013532	1206806	49.98	0.1593	345	-	0.1500	-	-	-	-	-	0.2214	-	-0.1489	-	-	-	-	-	-	-	-	-	0.1623
Chlamydophila pneumoniae TW 183 uid57997	1225935	40.58	NC_005043	L	W	NC_010287	1038842	41.33	0.2162	560	-	-	-	-	0.2079	-0.1189	-	-	-	-0.1116	-0.1585	-	-	-0.0978	0.1499	-	0.1056	-	-	-
Candidatus Blochmannia floridanus uid57999	705557	27.38	NC_005061	I	W	NC_004344	697724	22.48	0.3839	302	-	-	0.2132	-	0.1752	-	0.1206	-0.1138	-	-0.1823	-0.1521	-	-	-	-	-0.1906	0.1003	-	-	-0.1258

Chromobacterium violaceum ATCC 12472 uid58001	4751080	64.83	NC_005085	A	C	NC_012559	3169329	62.35	0.3734	1298	-0.0987	-0.0769	-0.2017	-	0.2183	-0.0486	-	0.1064	-	-	-0.1208	-0.0701	-0.0969	-	-	-	-	-	0.1161	-0.0773
Ehrlichia ruminantium Welgevonden uid58013	1516355	27.48	NC_005295	I	W	NC_007354	1315030	28.96	0.3339	635	-	-	-	-	-	0.0752	-	-0.2713	-	-0.2134	-0.0984	-0.1371	0.1423	-0.1913	-	-0.1348	-	-	-0.0761	-
Candidatus Protochlamydia amoebophila UWE25 uid58079	2414465	34.72	NC_005861	L	W	NC_015702	3072383	39.03	0.2495	717	0.0971	-	0.0928	-	-	-	-	-	0.1913	-0.1508	-0.1118	-0.1238	-	-0.1583	-	-	0.1777	-	-	-
Bacillus anthracis Sterne uid58091	5228663	35.38	NC_005945	L	C	NC_008600	5257091	35.43	0.0606	2009	-	-	0.0648	-	-	-	-	-0.0957	-	-0.0761	-0.0783	0.0849	-	-0.0834	-	-	-	-	0.0890	-
Bartonella quintana Toulouse uid57635	1581384	38.80	NC_005955	L	W	NC_014932	1522743	35.73	0.2772	824	0.1258	-	-0.0824	-	0.1335	-	-	-0.1584	-	-0.2314	-0.1684	-	-	-0.1665	0.1189	-0.1364	0.0984	-	-	-
Bartonella henselae Houston 1 uid57745	1931047	38.23	NC_005956	L	W	NC_012846	2341328	38.06	0.2204	1088	0.1310	0.0572	-	0.1009	-	-	0.1001	-0.0647	0.0725	-0.1806	-0.0876	-	-	-0.1810	0.1244	-0.0873	0.1477	-	-	-
Acinetobacter ADP1 uid61597	3598621	40.43	NC_005966	L	C	NC_011586	4050513	39.21	0.1693	1765	0.1091	-	0.0577	-	0.0655	-	-	-0.0573	-	-0.1621	-0.1243	-0.1452	-	-0.1426	0.0969	-	0.0952	0.0787	-	0.0568
Bacillus licheniformis ATCC 14580 uid58097	4222597	46.19	NC_006270	L	C	NC_006582	4303871	44.75	0.1550	1282	-	-	0.0847	-0.0551	0.0989	-	-	0.0574	0.1543	-0.1129	-	-	-	-0.1177	0.1344	-	-	-	-	-
Bacillus licheniformis ATCC 14580 uid58199	4222645	46.19	NC_006322	L	C	NC_006582	4303871	44.75	0.1527	1305	-	-	0.0711	-0.0579	0.1104	-	-	-	0.1327	-0.1124	-	-	-	-0.1166	0.1308	-	-	-	-	-
Bacillus clausii KSM K16 uid58237	4303871	44.75	NC_006582	L	C	NC_006322	4222645	46.19	0.1976	1305	-0.1187	-	-	-	0.1477	-0.0952	0.0561	0.0739	0.0701	-0.1476	-0.1442	-	-	-0.1110	0.0585	-	0.0790	0.1398	0.0711	-
Ehrlichia ruminantium Gardel uid58245	1499920	27.51	NC_006831	I	W	NC_007354	1315030	28.96	0.3176	546	-	0.0868	-	-	0.0938	-	-	-0.2171	-	-0.2386	-	-0.1097	0.1483	-0.1182	-	-0.1134	-	-	-	-
Ehrlichia ruminantium Welgevonden uid58243	1512977	27.48	NC_006832	I	W	NC_007354	1315030	28.96	0.3288	543	-	0.0978	-	-	-	-	-	-0.2377	-	-0.2192	-	-0.1538	0.1610	-0.1344	-	-0.1171	-	-	-	-
Corynebacterium glutamicum ATCC 13032 uid61611	3282708	53.84	NC_006958	A	C	NC_015859	3433007	67.15	0.1744	718	-0.1051	-	-	-	0.0972	-0.1033	-	0.1440	-	-	-	-	-0.2025	-	0.0719	0.0933	-	-	0.1363	-
Candidatus Pelagibacter ubique HTCC1062 uid58401	1308759	29.68	NC_007205	K	W	NC_015975	2066652	43.47	0.3271	199	-	-0.1496	0.1787	-	-	-	-	-	0.3823	-0.2391	-	-	-	-	-0.1609	-0.1609	-	0.1467	-	-
Candidatus Blochmannia pennsylvanicus BPEN uid58329	791654	29.56	NC_007292	I	W	NC_004344	697724	22.48	0.3852	322	-	-	0.2234	-	0.1020	-	-	-0.1976	-	-0.1945	-	-0.1428	-	-0.1365	0.1321	-0.1119	-	-	0.1119	-0.1001
Dechloromonas aromatica RCB uid58025	4501104	59.25	NC_007298	A	C	NC_008702	4376040	67.92	0.2454	1656	-0.0833	-0.0480	-0.1429	-0.0627	0.1733	-0.0480	-	0.1379	-	-0.0548	-	-	-0.1485	-	0.0674	0.0788	-	-	0.1112	-
Ehrlichia canis Jake uid58071	1315030	28.96	NC_007354	I	W	NC_007799	1176248	30.10	0.2897	669	0.0909	-	-	-	-	0.0906	0.0894	-0.1638	0.1027	-0.1720	-	-0.0957	0.2343	-0.1563	-	-0.0913	-	-	-0.1341	0.0760
Dehalococcoides CBDB1 uid58413	1395502	47.03	NC_007356	L	W	NC_009455	1341892	47.17	0.0238	598	-	-	-	-	0.1220	-	-	-	-	-	-	-	-	-	-	-	-	-	-	-
Carboxydothermus hydrogenoformans Z 2901 uid57821	2401520	42.05	NC_007503	L	W	NC_014209	2306092	34.32	0.2124	576	-	-	-	-	0.2993	-	0.1747	-	-	-0.1765	-0.1093	-	-	-0.1794	-	-0.0789	-	0.1650	0.0757	-
Chlorobium luteolum DSM 273 uid58175	2364842	57.33	NC_007512	A	W	NC_009454	3025375	52.96	0.2400	289	-0.1275	-0.1382	-	-	0.2453	-	-	-	-	-	-	-	-	-	-	-	-0.1313	0.1156	-	-0.1104
Chlorobium chlorochromatii CaD3 uid58375	2572079	44.28	NC_007514	L	W	NC_011027	2289249	55.80	0.2666	1138	-	-	-0.1416	-	0.2090	-	-	0.1236	-0.0863	-0.1602	-0.1928	-	-	-0.0697	0.0709	-	0.1468	0.0973	0.0811	-
Desulfovibrio alaskensis G20 uid57941	3730232	57.84	NC_007519	A	W	NC_011769	4040304	67.11	0.3175	1530	-0.1480	-	-0.1783	-0.1374	0.0942	-	-	0.2112	-	-0.0900	-0.0763	-0.0686	-0.1289	-	-	-	0.0921	-	0.0499	-
Erythrobacter litoralis HTCC2594 uid58299	3052398	63.07	NC_007722	A	C	NC_015976	4199332	65.57	0.2779	1054	-0.1758	-	-0.0899	-0.0883	-	-0.0633	-	0.2532	-	-	-0.0756	-	-0.1076	-	-	0.1242	-	-	0.0988	-
Anaeromyxobacter dehalogenans 2CP C uid58135	5013479	74.91	NC_007760	A	C	NC_011145	5061632	74.84	0.2219	2449	-0.0715	-0.0707	-0.1115	-0.0513	-0.1016	0.0453	-	0.1864	-0.0629	-	-0.0438	-	-0.1003	-	-	0.1319	-	-0.0819	0.1011	0.0531
Anaplasma phagocytophilum HZ uid57951	1471282	41.64	NC_007797	L	W	NC_012026	1202435	49.77	0.1875	264	-	-	-	-	0.1906	-	-	-	-	-	-	-	-	-0.1429	0.1456	-0.1707	-	-	0.1511	-
Ehrlichia chaffeensis Arkansas uid57933	1176248	30.10	NC_007799	I	W	NC_007354	1315030	28.96	0.2478	669	-	-	-0.1120	-	-	-	0.1360	-0.1817	0.0932	-0.1912	-	-0.1034	0.1793	-0.1655	-	-0.0807	-	-	-0.1400	-
Chromohalobacter salexigens DSM 3043 uid62921	3696649	63.91	NC_007963	A	C	NC_014532	4061296	63.61	0.2904	1889	-0.1545	-0.0523	-0.1414	-0.1326	0.1392	-	-	0.0935	-0.1170	-	-	-0.0971	-0.0493	-	0.0948	-	0.0655	-	0.1197	-
Candidatus Koribacter versatilis Ellin345 uid58479	5650368	58.38	NC_008009	A	C	NC_011601	4965553	50.57	0.1378	528	-	-	-	-0.1029	0.1556	-	-	0.1323	-	-	-	-0.1107	-	-	-	-	-	0.1588	-	-
Escherichia coli 536 uid58531	4938920	50.52	NC_008253	L	C	NC_011740	4588711	49.94	0.0689	2312	-	0.0531	-	-	-	-0.0657	0.0907	-	-	-0.1238	-0.0427	-0.1000	-	-	0.0967	-	0.0901	-	0.0630	0.0391
Cytophaga hutchinsonii ATCC 33406 uid57651	4433218	38.85	NC_008255	L	W	NC_014655	4059653	40.41	0.2923	1006	-	-0.0761	0.0674	-	0.1991	-	-	-	0.1260	-0.2071	-0.1488	-0.1588	-	-0.1264	-	-	-	0.1269	-	0.0700
Clostridium perfringens ATCC 13124 uid57901	3256683	28.38	NC_008261	I	W	NC_010723	3659644	27.36	0.2492	1220	-	-0.0682	0.0861	0.0911	-	0.0530	-	-0.1787	-	-0.1157	-0.1028	-	-	-0.1593	-	-0.2127	-	-	0.0981	-0.0605
Alkalilimnicola ehrlichii MLHE 1 uid58467	3275944	67.53	NC_008340	A	C	NC_011901	3464554	65.06	0.2825	1082	-0.1514	-	-0.1298	-0.1578	0.1381	-	-	-	-0.0594	-	-0.0729	-	-0.1103	-	-	-	-	0.0733	0.1715	-
Candidatus Solibacter usitatus Ellin6076 uid58139	9965640	61.90	NC_008536	A	C	NC_008009	5650368	58.38	0.1413	1269	-0.0834	-	-0.1286	-	0.1203	-	-	0.1178	-	-0.0774	-	-	-0.1160	-	-	-	-	0.1232	0.0885	-

Aeromonas hydrophila ATCC 7966 uid58617	4744448	61.55	NC_008570	L	C	NC_009348	4702402	58.51	0.1039	2618	0.0914	-	-0.1526	-0.0646	-	-	0.0660	-	-0.0856	-0.0762	-0.0801	-0.0822	-0.0813	-	-	-	-	-	0.0981	-0.0553
Acidothermus cellulolyticus 11B uid58501	2443540	66.91	NC_008578	A	C	NC_011896	3268071	57.80	0.3893	194	-0.3044	-	-	-0.1332	-	-	-	0.2017	-	-	-0.2117	-0.1748	-	-	-	-	-	-	0.1823	-
Clostridium novyi NT uid58643	2547720	28.86	NC_008593	K	W	NC_015425	2773157	28.49	0.2507	1367	0.1593	-	0.1033	0.1501	-	0.1195	-	-0.1088	-	-0.0949	-0.0868	-	0.0875	-	-	-0.1598	-	-	0.0814	-0.0614
Campylobacter fetus 82 40 uid58545	1773615	33.31	NC_008599	L	W	NC_003912	1777831	30.31	0.2757	812	0.1570	-	0.1299	0.1204	0.1083	-	-	-0.0966	0.0976	-0.1714	-	-	-	-0.1925	-	-0.1200	-	0.0835	-	-
Candidatus Ruthia magnifica Cm Calyptogena magnifica uid58645	1160782	34.03	NC_008610	L	W	NC_014774	1258278	35.24	0.1749	236	-	-	-	0.1969	-	-	-	-	-	-0.1872	-	-	-0.1730	-	-	-	-	-	0.2319	-
Bifidobacterium adolescentis ATCC 15703 uid58559	2089645	59.18	NC_008618	A	C	NC_014638	2214656	62.67	0.2709	738	-0.1804	-0.1114	-0.1413	-	0.1363	-	0.0954	0.1457	-0.0774	-	-	-0.1089	-0.0766	-	0.1411	-	-	-	0.0949	-
Chlorobium phaeobacteroides DSM 266 uid58133	3133902	48.35	NC_008639	L	W	NC_011060	3018238	48.08	0.1022	1520	-	-	-0.1027	-	0.1268	-0.0624	0.0637	0.0785	0.0780	-0.0856	-0.0837	-	-0.0758	-	0.1114	-	-	-	0.0650	-
Borrelia turicatae 91E135 uid58311	917330	29.12	NC_008710	I	W	NC_010673	922307	29.83	0.2871	518	0.1258	-0.0910	-	0.1897	0.1154	-	0.0923	-0.1402	-	-0.1531	-0.1057	-0.1038	-	-	-	-0.0887	-	-	-	-
Acidovorax citrulli AAC00 1 uid58429	5352772	68.53	NC_008752	A	C	NC_015138	5482170	68.82	0.1944	3054	-	0.0390	-0.0788	-0.0511	-0.0695	-	0.0754	0.1389	-0.0456	-	-	-0.0486	-0.0575	0.0581	0.1100	0.1575	-	-0.0738	0.0979	-
Bartonella bacilliformis KC583 uid58533	1445021	38.24	NC_008783	L	W	NC_005955	1581384	38.80	0.2699	804	0.1392	-	-	-	-	-	-	-0.1100	-	-0.2291	-0.1391	-	-	-0.1740	0.1153	-0.1518	0.1415	-	0.0860	-
Clostridium thermocellum ATCC 27405 uid57917	3843301	38.99	NC_009012	I	W	NC_011837	3896121	31.99	0.1977	597	-	-	0.1285	0.0957	0.1582	-	0.1218	-0.1143	-	-	-0.1135	-	0.1243	-0.1063	0.0918	-0.1477	-	0.1529	-	-
Actinobacillus pleuropneumoniae serovar 5b L20 uid58789	2274482	41.30	NC_009053	L	C	NC_009655	2319663	44.92	0.2143	1311	-	-	-	-	0.1641	-0.0732	-	0.0615	0.1262	-0.1406	-0.1054	-	-	-0.1494	0.0565	-0.0579	0.0970	0.0844	0.0606	-
Desulfotomaculum reducens MI 1 uid58277	3608104	42.28	NC_009253	L	W	NC_015573	3601386	54.88	0.1593	674	-	-	-	-	0.2536	-	-	-	-	-	-	-	-	-0.1066	-	-	0.0921	0.1679	0.1303	-
Chlorobium phaeovibrioides DSM 265 uid58129	1966858	52.99	NC_009337	L	W	NC_010831	2736403	48.93	0.2512	1153	-0.0899	-	-0.1670	-0.1015	0.1794	-	-	0.1405	-	-	-	-	-0.1380	-	0.1292	0.0756	-	-	-	-
Caldicellulosiruptor saccharolyticus DSM 8903 uid58289	2970275	35.25	NC_009437	I	W	NC_014652	2770676	36.11	0.2163	1371	0.0698	-0.0710	-	0.0712	0.1009	-	0.0696	-0.1238	0.1602	-0.1032	-	-	-	-0.0593	0.0691	-0.1221	-	-	0.0699	-0.0735
Bradyrhizobium ORS 278 uid58941	7456587	65.51	NC_009445	A	C	NC_009485	8264687	64.92	0.2347	3851	-0.0853	0.0336	-0.1328	-0.1011	-	-0.0333	-	0.1807	-0.0708	-	-	-0.1133	-0.0602	0.1205	0.1227	0.1461	0.0325	-0.0424	0.0603	-
Dichelobacter nodosus VCS1703A uid57643	1389350	44.40	NC_009446	A	W	NC_008610	1160782	34.03	0.2020	360	-	-	0.2008	-	0.2454	0.0999	-	0.1591	-	-	-	-	-	-	-	-	-	0.1521	0.2287	-
Dehalococcoides BAV1 uid58477	1341892	47.17	NC_009455	L	W	NC_013552	1413462	47.27	0.1208	920	0.1186	-	-0.0885	-	0.2316	-0.1413	-	-	-	-0.0867	-0.0998	-0.1282	-	-	-	0.0708	-	-	-	-
Candidatus Vesicomyosocius okutanii HA uid59427	1022154	31.59	NC_009465	I	W	NC_015722	1183732	36.55	0.1844	239	-	-	-	-	-	-	-	-	-	-0.2608	-	-0.2961	-	-	-	-	-	0.1386	-	-
Bacteroides vulgatus ATCC 8482 uid58253	5163189	42.20	NC_009614	L	W	NC_006347	5277274	43.27	0.0870	2017	-	-0.0615	-	-	0.0786	-	0.1023	-0.0599	-	-0.1236	-0.1005	-	0.0687	-	0.0922	-0.0713	-	0.0543	0.0468	-
Clostridium beijerinckii NCIMB 8052 uid58137	6000632	29.86	NC_009617	I	W	NC_014393	5262222	31.21	0.2162	1378	0.0914	-0.1173	0.1592	0.0973	-	0.0708	0.0489	-0.1317	0.1445	-0.1234	-	-	0.0630	-0.0815	-	-0.1109	-	0.0654	-	-
Alkaliphilus metalliredigens QYMF uid58171	4929566	36.82	NC_009633	L	W	NC_013851	3526903	64.37	0.2144	418	-	-	0.2233	0.1003	0.1362	0.0902	-	-0.1505	-	-	-	-	-	-0.1485	-	-0.1387	-	0.1084	-	-
Actinobacillus succinogenes 130Z uid58247	2319663	44.92	NC_009655	L	C	NC_009053	2274482	41.30	0.1945	1311	-	-	-	-	0.1681	-0.0546	-	-	0.1176	-0.2027	-0.0732	-0.1119	-	-0.0975	-	-	0.0969	0.1044	0.0659	0.0560
Anaeromyxobacter Fw109 5 uid58755	5277990	73.53	NC_009675	A	C	NC_011145	5061632	74.84	0.2968	1713	-0.1379	-0.0662	-0.1550	-0.1095	-	-	0.0429	0.1949	-0.0648	-	-	-0.0897	-0.0500	-	0.0809	0.0656	-	-	0.1224	-
Clostridium botulinum A ATCC 19397 uid58927	3863450	28.21	NC_009697	I	W	NC_011837	3896121	31.99	0.2825	906	-	-	0.1643	0.0749	-	0.1513	0.1071	-0.1802	-	-0.1280	-	-	0.1032	-0.1384	-	-0.1814	-	-	-	-
Clostridium botulinum A Hall uid58931	3760560	28.18	NC_009698	I	W	NC_011837	3896121	31.99	0.2822	901	-	-	0.1644	0.0776	-	0.1478	0.1070	-0.1832	-	-0.1319	-	-	0.1016	-0.1375	-	-0.1757	-	-	-	-
Campylobacter jejuni doylei 269 97 uid58671	1845106	30.57	NC_009707	L	W	NC_008599	1773615	33.31	0.3574	757	0.1334	-0.1349	-	0.0946	0.1338	0.0743	0.0687	-0.0916	0.1289	-0.1407	-0.1001	-	-	-0.2131	-	-0.1555	-	-	-	-
Campylobacter curvus 525 92 uid58669	1971264	44.54	NC_009715	L	W	NC_009802	2052007	39.43	0.2119	981	-	-0.0928	-0.0911	-	0.0836	-0.0814	-	-	0.2508	-0.1254	-0.0816	-	-	-0.1333	-	-0.1437	-	-	-	-0.1039
Bacillus amyloliquefaciens FZB42 uid58271	3918589	46.48	NC_009725	L	C	NC_002570	4202352	43.69	0.2220	1208	-	-	-	-0.0581	0.1295	-	-	0.0672	0.0665	-0.1689	-0.0929	-0.1726	-	-0.1569	0.1204	-0.0608	-	-	-	0.0725
Escherichia coli HS uid58393	4643538	50.82	NC_009800	L	C	NC_011740	4588711	49.94	0.0564	2244	-	-	-	-	-	-0.0682	0.1068	-	-	-0.1280	-	-0.0887	-	-	0.0911	-	0.0758	-	0.0465	-
Campylobacter jejuni 81116 uid58771	1628115	30.54	NC_009839	L	W	NC_012039	1525460	29.70	0.3491	1006	0.1443	-0.0714	0.1118	0.1318	0.1491	0.0648	-	-0.1058	0.1186	-0.1775	-	-0.1262	-	-0.1365	-	-0.1128	0.0917	-	-	-
Bacillus pumilus SAFR 032 uid59017	3704465	41.29	NC_009848	L	W	NC_014219	3592487	48.67	0.2028	980	0.1106	-	0.1167	-	0.1486	-	-	-	0.0986	-0.1481	-	-0.0917	-	-0.1782	0.0901	-0.0858	0.0998	0.1375	0.0954	0.0962
Arcobacter butzleri RM4018 uid58557	2341251	27.05	NC_009850	K	W	NC_014166	3192235	28.36	0.2774	1188	0.1431	-0.1348	-	-	-	0.0958	-	-0.1112	0.0820	-0.2120	-0.1239	-	-	-0.0974	-	-0.1502	-	-	-	-

Alkaliphilus oremlandii OhILAs uid58495	3123558	36.26	NC_009922	I	W	NC_013851	3526903	64.37	0.2221	337	-	-	0.2237	-	0.1257	-	-	-0.1117	-	-	-0.1101	-	-	-0.2322	-	-0.1589	-	0.1583	-	-
Azorhizobium caulinodans ORS 571 uid58905	5369772	67.32	NC_009937	A	C	NC_013854	3311395	67.76	0.2378	771	-	-0.0696	-0.1493	-0.1160	0.1447	-	-	0.1826	-	-	-0.0836	-	-	0.0755	0.0938	0.0952	-	-	0.0894	-0.0785
Desulfococcus oleovorans Hxd3 uid58777	3944167	56.17	NC_009943	A	W	NC_014972	3851869	58.93	0.1666	768	-	-	-0.0757	-0.0931	0.2162	-	0.0796	0.1008	0.0759	-0.1316	-0.0814	-	-	-	-	-0.0998	-	0.1591	-	-
Clostridium phytofermentans ISDg uid58519	4847594	35.35	NC_010001	L	W	NC_014376	4662871	45.00	0.1803	1315	-	-	0.0967	-	0.1325	-	0.0952	-	0.0715	-0.1597	-0.1133	-0.0786	0.0792	-0.0873	-	-0.1762	-	0.0816	-	-
Delftia acidovorans SPH 1 uid58703	6767514	66.48	NC_010002	A	C	NC_015563	6685842	66.72	0.0817	3428	-0.0723	-	-0.0395	-	-0.0401	-	0.0654	0.0473	-0.0357	-	-	-0.0405	-0.0749	-	0.0591	0.0958	0.0389	-0.0683	0.0919	-
Acholeplasma laidlawii PG 8A uid58901	1496992	31.93	NC_010163	K	C	NC_010544	879959	27.42	0.3153	202	-	-	0.2548	-	0.2476	0.1677	-	-	-	-0.2259	-	-	-	-	-	-0.2035	-	0.1523	-	-
Bordetella petrii DSM 12804 uid61631	5287950	65.48	NC_010170	A	C	NC_002928	4773551	68.10	0.2999	2023	-0.1203	-	-0.1273	-0.1153	0.0821	-0.0408	-	0.1693	-0.0953	-	-0.0936	-0.1039	-	-	0.0541	0.0785	0.0507	-	0.0820	-
Chloroflexus aurantiacus J 10 fl uid57657	5258541	56.70	NC_010175	L	C	NC_011831	4684931	56.43	0.2916	2712	-0.1367	-	-	-0.0866	0.1269	-0.1031	-	-	-0.1572	-0.1020	-0.0384	-0.1507	-0.1014	-	0.0694	0.1127	0.1235	0.0364	0.1527	0.0687
Actinobacillus pleuropneumoniae serovar 3 JL03 uid58891	2242062	41.23	NC_010278	L	C	NC_009655	2319663	44.92	0.2067	1281	-	-	-	-	0.1461	-0.0715	-	-	0.0830	-0.1207	-0.1179	-0.0684	-	-0.1709	0.0682	-0.0570	0.1182	-	0.0552	-
Chlamydia trachomatis L2b UCH 1 proctitis uid61635	1038863	41.33	NC_010280	L	W	NC_005043	1225935	40.58	0.2082	559	-	-	-	-0.1247	0.1368	-0.0855	0.1061	-	-	-0.1075	-0.1649	-	-	-	0.2161	-0.0927	0.0909	-	-	-
Chlamydia trachomatis 434 Bu uid61633	1038842	41.33	NC_010287	L	W	NC_005043	1225935	40.58	0.2092	560	-	-	-	-0.1298	0.1217	-0.0852	0.0875	-	-	-0.0912	-0.1659	-	-	-	0.2225	-0.0965	0.1030	-	-	-
Candidatus Desulforudis audaxviator MP104C uid59067	2349476	60.85	NC_010424	L	W	NC_014964	2344824	34.50	0.2200	338	-0.1570	-	-	-	0.2329	-	-	-	-	-	-0.1104	-	-0.1293	-	-	-	-	0.1965	0.1932	-
Candidatus Phytoplasma australiense uid61641	879959	27.42	NC_010544	K	W	NC_005303	853092	27.76	0.4077	222	-	-	0.1163	0.1793	0.1311	-	-	-	0.3281	-0.1660	-	-	-0.1539	-	-	-0.1771	-	-	0.1225	-
Corynebacterium urealyticum DSM 7109 uid61639	2369219	64.19	NC_010545	A	C	NC_014329	2337913	52.19	0.2129	623	-0.1439	-	-	-0.0813	-	-	-	0.2196	-0.0973	-	-0.0847	-0.0923	-0.0926	-	-	-	-	0.1209	0.1026	-
Bordetella avium 197N uid61563	3732255	61.58	NC_010645	A	C	NC_002929	4086189	67.72	0.2652	1669	-0.0858	-	-0.1413	-0.0854	0.1117	-0.0916	-	0.1615	-0.1023	-	-0.1289	-0.0912	-0.0598	-	0.0454	0.0787	-	-	-	-0.0598
Akkermansia muciniphila ATCC BAA 835 uid58985	2664102	55.76	NC_010655	L	W	NC_010794	2287145	45.48	0.2712	350	-0.1243	-	-	-	0.3016	-	0.1148	-	-	-	-0.2314	-	-0.1013	-	-	-	-	0.2160	0.1392	-
Borrelia hermsii DAH uid59225	922307	29.83	NC_010673	I	W	NC_008710	917330	29.12	0.2592	518	-	-	-	0.1491	0.1245	-	-	-0.1466	-	-0.1776	-0.1257	-0.1104	-	-	-	-0.0999	-	-	-	-
Clostridium botulinum E3 Alaska E43 uid59157	3659644	27.36	NC_010723	I	W	NC_009012	3843301	38.99	0.2953	680	-	-	0.1493	-	-	-	-	-0.2007	-	-0.0925	-0.0887	-	0.0816	-0.2185	-	-0.2066	-	-	-	-
Chlorobium limicola DSM 245 uid58127	2763181	51.32	NC_010803	L	W	NC_007514	2572079	44.28	0.2017	1183	-0.0952	-0.0623	-0.1680	-0.0784	0.2300	-0.0578	-	-	-	-0.1187	-0.0968	-	-0.1119	-	0.1080	-	-	-	-	-
Candidatus Amoebophilus asiaticus 5a2 uid58963	1884364	35.05	NC_010830	L	W	NC_014644	1667350	41.36	0.1597	102	-	-	-	-	-	-	-	-	-	-	-	-	0.1928	-	-	-	0.2559	-	-	-
Chlorobium phaeobacteroides BS1 uid58131	2736403	48.93	NC_010831	L	W	NC_009337	1966858	52.99	0.1732	1153	-0.1342	-	-0.1286	-	0.2440	-0.0867	0.0640	-	-	-	-0.0789	-	-	-	0.1360	-	0.0789	0.1635	-	-
Cellvibrio japonicus Ueda107 uid59139	4576573	51.99	NC_010995	L	C	NC_015556	4920769	63.51	0.2403	1011	-	-	-	-	0.2473	-0.1058	-	0.1342	-	-	-	-0.0900	-	-	0.0924	-	0.1443	0.1322	0.1465	-
Chloroherpeton thalassium ATCC 35110 uid59187	3293456	45.04	NC_011026	L	W	NC_011831	4684931	56.43	0.1369	538	-	-	-	-	0.1929	-0.1083	-	0.1110	-	-0.1175	-	-	-	-0.1226	0.1217	-	-	0.1584	-	-
Chlorobaculum parvum NCIB 8327 uid59185	2289249	55.80	NC_011027	L	W	NC_007514	2572079	44.28	0.2067	1138	-0.0746	-0.0902	-0.1758	-0.1128	0.0888	-0.0617	-	0.0983	-	-0.1343	-0.0662	-0.0804	-0.1400	-	0.1128	-	-	-	0.1148	-0.0668
Candidatus Phytoplasma mali uid59087	601943	21.39	NC_011047	K	W	NC_007716	706569	26.89	0.4237	209	-	-	0.1571	-	-	0.2343	0.1824	-0.2151	0.1468	-0.2602	-	-	-	-	-	-0.1883	-	-	-0.1378	-
Alteromonas macleodii Deep ecotype uid58251	4448980	44.89	NC_011138	L	C	NC_015554	4972148	43.51	0.1461	2037	0.0630	-	-	0.0458	-	-0.0705	-	0.0490	-	-0.1950	-0.1393	-0.0937	-	-0.1086	0.1496	-	-	0.0549	-	0.0756
Anaeromyxobacter K uid58953	5061632	74.84	NC_011145	A	C	NC_011891	5029329	74.72	0.1265	2469	-0.0506	-0.0380	-0.0587	-0.1115	-0.0482	-	0.0565	0.0805	-0.0914	-	-0.0632	-0.0656	-	0.0612	-	0.0779	-	-0.0764	0.0547	0.0995
Acidithiobacillus ferrooxidans ATCC 53993 uid58613	2885038	58.85	NC_011206	A	C	NC_015850	2932225	61.32	0.3487	1015	-0.1486	-	-0.1595	-0.0592	0.1562	-0.0744	-	0.1199	-0.1667	-	-0.1253	-0.1496	-0.0893	-	-	0.0696	-	-	0.1604	-
Coprothermobacter proteolyticus DSM 5265 uid59253	1424912	44.77	NC_011295	L	W	NC_015499	1898865	33.87	0.1814	223	-	-	-	-	0.1684	-	-	-	-	-0.1372	-	-	-	-	-	-	-	0.2109	0.1590	-
Dictyoglomus thermophilum H 6 12 uid59439	1959987	33.74	NC_011297	L	C	NC_011661	1855560	33.96	0.3634	1317	0.0982	-0.1025	-	0.1314	-	-0.0520	-	-0.2446	0.0990	-	-	-	0.0894	-0.0763	-	-0.1823	-0.0854	-0.0661	-	-0.1294
Coxiella burnetii CbuG Q212 uid58893	2008870	42.63	NC_011527	L	W	NC_006369	3345687	38.41	0.2949	553	-	-	0.1560	-	0.1680	0.1077	-	-	-	-0.1411	-0.1022	-0.1185	-0.1058	-	0.1291	-	-	0.1369	0.2009	-
Anoxybacillus flavithermus WK1 uid59135	2846746	41.78	NC_011567	L	C	NC_016047	4207222	43.82	0.2135	873	-0.0893	-	-	-0.1020	-	-	-	-	-	-0.1634	-0.1080	-0.1390	-	-0.1238	-	-0.1060	-	0.1567	0.1110	0.0680
Bifidobacterium longum infantis ATCC 15697 uid58677	2832748	59.86	NC_011593	A	C	NC_013714	2636367	58.54	0.2212	855	-0.1424	-	-0.0804	-	0.0924	-	0.0946	0.1387	-0.1196	-	-	-0.1689	-0.0819	-	0.1199	-	0.0874	-	0.0898	-

Acinetobacter baumannii AB307 0294 uid59271	3760981	39.04	NC_011595	L	C	NC_014259	4152543	38.73	0.0842	2613	0.1200	-	0.0620	-	-	0.0642	0.0799	-	-	-0.1589	-0.0609	-	-	-0.0749	-	-0.0624	-	-	0.0551	-
Dictyoglomus turgidum DSM 6724 uid59177	1855560	33.96	NC_011661	L	C	NC_011297	1959987	33.74	0.3687	1317	0.1292	-0.1127	0.0773	0.1052	0.0632	-0.0682	-	-0.2137	0.1245	-	-	-	0.1383	-0.0622	-	-0.1742	-0.0683	-	-	-0.1002
Bacillus cereus B4264 uid58757	5419036	35.30	NC_011725	L	C	NC_012581	5230115	35.38	0.1590	3245	0.1156	0.0479	0.1232	0.0778	-	-	0.0595	-0.1143	-	-0.1348	-0.0847	-	0.0936	-0.0991	0.0605	-0.1066	-	-	0.0794	-
Escherichia coli ED1a uid59379	5209548	50.73	NC_011745	L	C	NC_011740	4588711	49.94	0.0420	2335	-	0.0484	-	-	-	-	0.0961	-	-	-0.0866	-	-0.0556	-	-	0.1001	-	0.0475	-	-	0.0552
Escherichia coli 55989 uid59383	5154862	50.66	NC_011748	L	C	NC_011740	4588711	49.94	0.0588	2311	-	0.0404	-0.0481	-	-	-	0.1020	0.0430	-	-0.1478	-	-0.1106	-	-	0.0812	-	-	-	0.0495	0.0459
Acidithiobacillus ferrooxidans ATCC 23270 uid57649	2982397	58.77	NC_011761	A	C	NC_015942	3207552	56.59	0.1323	1430	-	-	-0.0999	-	-	-	-	0.0912	-0.1006	-0.0861	-0.0678	-0.0921	-0.0599	-	0.0914	0.1106	-	-	0.1585	-
Desulfatibacillum alkenivorans AK 01 uid58913	6517073	54.48	NC_011768	A	W	NC_014365	3655731	65.70	0.0923	950	-	-	-	-	0.1912	-	-	-	0.0880	-	-	-	-0.0688	-	0.0791	-	-	0.0904	-	-0.0822
Desulfovibrio vulgaris Miyazaki F uid59089	4040304	67.11	NC_011769	A	W	NC_014844	3629109	62.56	0.2712	1214	-0.0747	-0.0919	-0.1645	-0.0868	0.1465	-	-	0.1680	-0.0563	-	-	-	-0.1080	-	-	-	0.0848	-	0.1653	-
Chloroflexus aggregans DSM 9485 uid58621	4684931	56.43	NC_011831	L	C	NC_010175	5258541	56.70	0.3074	2712	-0.1351	-	-	-0.0796	0.1454	-0.0943	0.0334	-	-0.1835	-0.1304	-0.0477	-0.1510	-0.0968	-	0.0619	0.1181	0.0913	0.0590	0.1551	0.1038
Buchnera aphidicola 5A Acyrthosiphon pisum uid59285	642122	26.29	NC_011833	I	W	NC_014909	722593	27.51	0.4553	310	0.1430	-	0.1769	-	0.2038	-	-	-0.1671	-	-0.3190	-0.1215	-	-	-0.2102	-	-	-	0.1423	-	-
Buchnera aphidicola Tuc7 Acyrthosiphon pisum uid59283	641895	26.29	NC_011834	I	W	NC_014909	722593	27.51	0.4384	308	0.1306	-	0.1863	-	0.1952	-	-	-0.1679	-	-0.3234	-0.1092	-	-	-0.1929	-	-	-	0.1396	-	-
Bifidobacterium animalis lactis AD011 uid58911	1933695	60.49	NC_011835	A	C	NC_014616	2186882	62.76	0.1717	726	-0.1501	-	-0.1102	-	-	-	0.1304	0.1390	-	-0.0955	-0.0976	-0.1098	-0.0751	0.0773	-	-	-	-	-	-
Desulfovibrio desulfuricans ATCC 27774 uid59213	2873437	58.07	NC_011883	A	W	NC_002937	3570858	63.14	0.2759	1058	-0.1730	-	-0.1646	-	0.1794	-	-	0.1178	-0.0766	-0.0597	-0.1194	-	-0.1314	-	-	-	0.0619	0.0698	0.0979	-0.0604
Anaeromyxobacter dehalogenans 2CP 1 uid58989	5029329	74.72	NC_011891	A	C	NC_011145	5061632	74.84	0.1229	2469	-	-0.0453	-0.0503	-0.1019	-	-	0.0682	0.0911	-0.0874	-	-0.0436	-0.0770	-	-	-	0.0641	-	-0.0744	0.0550	0.0818
Clostridium cellulolyticum H10 uid58709	4068724	37.40	NC_011898	I	W	NC_012563	4155278	28.21	0.1938	749	-	-	0.1651	-	0.1019	-	-	-0.1147	-	-	-	-	0.1247	-0.1290	0.1169	-0.1461	-	-	0.1079	-
Acidovorax ebreus TPSY uid59233	3796573	66.83	NC_011992	A	C	NC_008782	4448856	66.17	0.0952	2088	-	-	-0.1105	0.0519	-	-	-	0.1461	-	-	-	-	-0.0473	0.0654	-	0.0642	-	-	0.0776	-
Anaplasma marginale Florida uid58577	1202435	49.77	NC_012026	V	W	NC_007797	1471282	41.64	0.2190	264	-0.1583	0.1236	-	-	0.1923	-	0.1554	0.2109	-	-	-	-	-	-	0.1328	-	-	-	-	-
Chloroflexus Y 400 fl uid59085	5268950	56.68	NC_012032	L	C	NC_011831	4684931	56.43	0.2870	2711	-0.1395	-	-	-0.0830	0.1230	-0.0941	-	-	-0.1540	-0.1113	-0.0491	-0.1736	-0.0968	-	0.0702	0.1198	0.1202	0.0521	0.1275	0.0668
Acidobacterium capsulatum ATCC 51196 uid59127	4127356	60.50	NC_012483	A	C	NC_015064	4309153	60.51	0.1586	1099	-0.0718	-	-0.0717	-	0.1624	-	-	0.1101	-0.0933	-	-0.1026	-0.1052	-	-	0.1070	0.0770	0.0681	-	0.0676	-
Brevibacillus brevis NBRC 100599 uid59175	6296436	47.27	NC_012491	L	C	NC_014622	5731816	45.24	0.1502	1368	-	-	-	-	0.0822	-	-	-	-	-0.0664	-0.1421	-0.1034	-	-0.0606	0.0812	-	-	0.1373	0.1169	-
Azotobacter vinelandii DJ uid57597	5365318	65.68	NC_012560	L	C	NC_015410	5434353	62.51	0.2602	1896	-0.0681	-	-0.1151	-0.0887	0.2139	-	-	0.0772	-	-	-0.0447	-0.0567	-0.1083	-	0.0652	0.0705	-	-	0.0979	-
Clostridium botulinum A2 Kyoto uid59229	4155278	28.21	NC_012563	I	W	NC_011898	4068724	37.40	0.3077	749	-	-0.0959	0.1293	-	0.0763	0.1777	-	-0.2190	-	-	-	-	0.0897	-0.1558	0.0797	-0.1309	-0.1094	-	-	-
Beutenbergia cavernae DSM 12333 uid59047	4669183	73.12	NC_012669	A	C	NC_014158	4379918	68.41	0.2298	537	-0.1094	-0.0933	-	-	-	-	-	0.1196	-	-	-	-	-0.1484	0.1261	-	-	-0.1292	-	0.1962	-
Chlamydia trachomatis B Jali20 OT uid59351	1044352	41.30	NC_012686	L	W	NC_015408	1106197	41.08	0.3042	589	-	-0.0862	-0.1616	-	0.1343	-0.0817	0.1802	-	-	-0.1638	-0.1528	-	-0.1032	-0.1429	0.2009	-	-	-	0.0885	-
Chlamydia trachomatis B TZ1A828 OT uid59349	1044282	41.30	NC_012687	L	W	NC_015408	1106197	41.08	0.3147	588	-	-	-0.1576	-	0.1514	-	0.1729	-	-	-0.1532	-0.1527	-	-0.1171	-0.1395	0.2085	-	-	-	0.0842	-
Burkholderia pseudomallei MSHR346 uid29855	4098576	67.71	NC_012695	A	C	NC_014722	2755309	61.21	0.3451	745	-0.0867	-	-0.1316	-0.1266	-	-0.0642	-	0.2738	-	-	-	-0.1255	-0.0896	-	-	-	-	-	0.1008	-0.0687
Corynebacterium kroppenstedtii DSM 44385 uid59411	2446804	57.46	NC_012704	A	C	NC_014329	2337913	52.19	0.3198	636	-0.1477	-	-	-	-	-	0.0675	0.1902	-	-	-0.0894	-0.1255	-0.1247	-	0.1978	-	-	0.1296	0.2175	-
Escherichia coli BW2952 uid59391	4578159	50.79	NC_012759	L	C	NC_011740	4588711	49.94	0.0590	2311	0.0637	-	-	-	-	-0.0547	0.0886	-	-	-0.1248	-0.0549	-0.0874	-	-	0.0777	-	0.0831	-0.0438	0.0740	-
Edwardsiella ictaluri 93 146 uid59403	3812315	57.44	NC_012779	L	C	NC_013508	3760463	59.73	0.1039	2092	-	0.0758	-	-	-	-	0.0573	-	-0.0581	-0.1313	-0.0501	-0.1228	-	0.0587	0.0532	-	-	-0.1144	0.0744	0.0721
Bifidobacterium animalis lactis Bl 04 uid59359	1938709	60.48	NC_012814	A	C	NC_014616	2186882	62.76	0.1857	750	-0.1323	-	-0.1549	-	0.1211	-	0.0854	0.1591	-	-0.1046	-	-0.1349	-0.0771	-	-	-	-	-	-	-
Bifidobacterium animalis lactis DSM 10140 uid59357	1938483	60.48	NC_012815	A	C	NC_014616	2186882	62.76	0.1858	748	-0.1319	-	-0.1552	-	0.1215	-	0.0847	0.1589	-	-0.1053	-	-0.1339	-0.0774	-	-	-	-	-	-	-
Dickeya dadantii Ech703 uid59363	4679450	55.02	NC_012880	L	C	NC_012912	4813854	54.52	0.1102	2612	-0.0508	-	-0.0789	-	-	-	-	0.0552	-	-0.1080	-0.0634	-0.0889	-0.0724	0.0468	0.1089	-	-	-0.0523	0.1346	-
Desulfovibrio salexigens DSM 2638 uid59223	4289847	47.09	NC_012881	L	W	NC_012796	5248049	62.77	0.1185	1271	-	-	-0.0608	-0.0643	0.1716	-0.0664	-	-	0.0973	-0.1039	-0.0660	-	-	-0.0800	0.1499	-	-	-	-	-0.0807

Dickeya zeae Ech1591 uid59297	4813854	54.52	NC_012912	L	C	NC_013592	4818394	53.64	0.0754	2898	-	-	-0.0746	-	-0.0743	-	-	0.0623	-	-0.1377	-	-0.0888	-0.0642	0.0565	0.0734	-	-	-0.0819	0.0837	0.0906
Aggregatibacter aphrophilus NJ8700 uid59407	2313035	42.23	NC_012913	L	C	NC_013416	2105764	44.55	0.1755	1336	0.0804	-	-	-	-	-	-	-	0.0934	-0.1639	-0.0727	-0.1149	-	-0.1203	-	-	0.2735	-	-	-
Escherichia coli B REL606 uid58803	4629812	50.77	NC_012967	L	C	NC_011740	4588711	49.94	0.0472	2257	-	-	-	-	-	-0.0754	0.0838	-	-	-0.1038	-	-0.1205	-	-	0.0833	-	-	-0.0613	-	-
Dyadobacter fermentans DSM 18053 uid59049	6967790	51.54	NC_013037	L	C	NC_015703	6568739	46.59	0.1112	2394	-	-0.0866	-	-0.0856	0.1680	-	-	0.0662	-	-0.1699	-	-0.0631	-	-	0.0835	-	-	0.0895	0.1020	-
Acidimicrobium ferrooxidans DSM 10331 uid59215	2158157	68.29	NC_013124	A	C	NC_014169	2477838	59.81	0.2802	164	-0.2260	-	-	-	0.2189	-	-	-	-	-	-	-	-	0.1988	-	-	-	0.2138	0.1705	-
Catenulispora acidiphila DSM 44928 uid59077	10467782	69.77	NC_013131	A	C	NC_003888	8667507	72.12	0.1549	1533	-0.1312	-	-	-0.1420	-	-	-	0.1570	-	-	-0.1111	-0.0819	-	0.0725	-	-	-	-	0.1085	-
Chitinophaga pinensis DSM 2588 uid59113	9127347	45.23	NC_013132	L	C	NC_009485	8264687	64.92	0.0792	630	-	-	-	-0.0987	0.1419	-	-	0.1194	-	-0.1088	-	-	-	-	-	-	-	0.0825	-	-
Capnocytophaga ochracea DSM 7271 uid59197	2612925	39.59	NC_013162	L	C	NC_015846	2571406	36.11	0.2306	1084	-	-0.1215	-	-	0.1455	-	0.0938	-	0.1364	-0.2514	-0.1141	-0.0908	-	-0.0996	-	-0.0904	0.0591	0.0815	0.1026	0.0846
Cryptobacterium curtum DSM 15641 uid59041	1617804	50.91	NC_013170	A	W	NC_013203	1543805	45.69	0.1636	389	-0.1584	-	-	-0.1266	-	-	-	0.1526	-	-	-0.1285	-	-	-	-	-0.1294	-	0.1228	-	-
Brachybacterium faecium DSM 4810 uid58649	3614992	72.05	NC_013172	A	C	NC_015588	3307740	73.89	0.2673	575	-0.1539	-0.1180	-0.1088	-0.1964	-	-	-	0.2204	-	-	-	-	-	0.1010	0.0814	-	-	0.0901	0.1042	-
Desulfomicrobium baculatum DSM 4028 uid59217	3942657	58.65	NC_013173	L	W	NC_012881	4289847	47.09	0.2168	1321	-	-	-0.1208	-0.0815	0.2687	-	0.0667	0.0785	-	-0.0924	-0.0790	-	-0.1627	-	0.0541	-	-	0.0824	0.0995	-
Atopobium parvulum DSM 20469 uid59195	1543805	45.69	NC_013203	A	W	NC_013170	1617804	50.91	0.1881	389	-	-	-	-0.1073	0.2411	-	0.1543	0.2335	-	-	-	-	-	-	-	-	-	0.1444	-	0.1147
Eggerthella lenta DSM 2243 uid59079	3632260	64.20	NC_013204	A	W	NC_015738	3123671	56.20	0.1686	1238	-	-	-0.1438	-0.0775	0.0998	-	0.0713	0.1521	-	-	-	-0.0867	-	-	0.1902	-	-	-	-	-
Desulfotomaculum acetoxidans DSM 771 uid59109	4545624	41.55	NC_013216	L	W	NC_009253	3608104	42.28	0.1419	714	-	-	-	-	0.1516	-	-	0.0909	-	-0.0926	-0.0840	-	-	-0.1120	0.1254	-	0.0942	0.0836	0.0794	-
Clostridium difficile CD196 uid41017	4110554	28.56	NC_013315	K	W	NC_012587	3925702	63.03	0.2357	348	-	-	0.1720	0.1269	-	-	-	-0.1174	-	-0.1718	-0.1775	-	-	-0.2125	-	-	-0.1372	-	-0.1113	-
Clostridium difficile R20291 uid40921	4191339	28.81	NC_013316	K	W	NC_011365	3887492	66.38	0.2543	317	-	-	0.1572	0.1592	-	-	0.1906	-0.1836	-	-	-0.1298	-	-	-0.2744	-	-	-	0.1164	-	-
Anaplasma centrale Israel uid42155	1206806	49.98	NC_013532	L	W	NC_004842	1197687	49.76	0.1564	345	-	0.1122	-	-	-	-	-	0.2321	-	-	-	-	-	-	-	-	0.1098	-	-	0.2016
Dehalococcoides VS uid42393	1413462	47.27	NC_013552	L	W	NC_009455	1341892	47.17	0.1403	920	0.1415	-0.0711	-0.0766	-	0.2228	-0.1343	-	-	-	-0.1113	-0.0700	-0.1542	0.0697	-	0.0975	-	-	-	-	-0.0655
Dickeya dadantii Ech586 uid42519	4818394	53.64	NC_013592	L	C	NC_012912	4813854	54.52	0.0744	2898	-	-	-0.0758	-	-0.0846	-	-	-	-	-0.1270	-0.0705	-0.1074	-0.0624	0.0472	0.0644	0.0460	-	-0.0858	0.0607	0.0829
Bifidobacterium dentium Bd1 uid43091	2636367	58.54	NC_013714	A	C	NC_010816	2375792	60.15	0.1822	851	-0.1383	-0.0770	-0.0813	-	-	0.0653	-	0.1563	-0.1555	-	-	-0.1705	-0.1440	-	-	-	-	-	0.0775	-
Conexibacter woesei DSM 14684 uid43467	6359369	72.73	NC_013739	A	C	NC_008726	6491865	67.79	0.1478	662	-0.2414	-	-	-0.0975	-	-	-	-	-0.1429	-	-0.0916	-	-	0.0960	-	-	-	-	-	-
Acidaminococcus fermentans DSM 20731 uid43471	2329769	55.84	NC_013740	L	W	NC_016077	2487765	50.02	0.1649	1017	-	-0.0924	-0.1259	-0.0912	0.1509	-	-	0.0727	-	-	-0.0779	0.1242	-	-	-	-	0.0983	-	0.1584	-
cyanobacterium UCYN A uid43697	1443806	31.12	NC_013771	L	C	NC_013161	4669813	39.82	0.3431	880	0.1511	-0.0739	0.1951	0.0762	0.1554	0.0983	-	-0.1224	0.1221	-0.1842	-0.1108	-	-0.0666	-0.0896	-	-0.0585	-	-	0.1050	-
Dehalococcoides GT uid42115	1360154	47.31	NC_013890	L	W	NC_002936	1469720	48.85	0.1098	836	-	-	-	-	0.2111	-0.0680	-	-0.1184	-	-0.0767	-0.0944	-	-	-0.0915	-	-	0.0702	-	0.0784	-
Clostridiales genomosp BVAB3 UPII9 5 uid46219	1809746	44.21	NC_013895	A	W	NC_012115	1676444	33.51	0.1888	163	-	-	-	-	0.2256	-	-	-	-	-0.1597	-0.1904	-	-	-	-	-	-	0.3330	-	-
Denitrovibrio acetiphilus DSM 12809 uid46657	3222077	42.54	NC_013943	L	W	NC_015672	2526590	38.29	0.1043	956	-	-0.0981	-	-	-	-	-	-	0.0880	-0.1904	-	-0.0847	-	-0.0888	0.0810	-0.1305	-0.0719	0.0708	-	-
Coraliomargarita akajimensis DSM 45221 uid47079	3750771	53.60	NC_014008	L	W	NC_010571	5957605	65.34	0.1964	938	-0.0969	-	-0.0651	-0.1205	0.2253	-	-	0.0793	-	-	-	-	-	-	0.0848	-	-	0.1398	0.1231	-
Candidatus Puniceispirillum marinum IMCC1322 uid47081	2753527	48.85	NC_014010	A	W	NC_008820	2682675	50.01	0.3384	280	-	-	0.1861	-0.1010	0.2549	-	-	0.2043	-	-	-	-0.2689	-	-	-	-	-	0.1237	-	-
Aminobacterium colombiense DSM 12261 uid47083	1980592	45.31	NC_014011	L	W	NC_013522	1848474	63.79	0.1524	458	-	-	-	-	-	-	-	-	-	-0.1331	-	-	-0.1339	-0.1360	0.1407	-	-	-	0.2189	-
Bacillus tusciae DSM 2912 uid48361	3384766	59.11	NC_014098	A	C	NC_013205	3018755	62.33	0.2534	438	-0.1883	-	-0.1301	-	0.1230	-	-	-	-	-0.1005	-	-0.1010	-	-	-	-	-	0.1301	0.2432	-0.1426
Bacillus megaterium DSM319 uid48371	5097447	38.13	NC_014103	L	C	NC_014171	5330088	35.29	0.1887	1758	-	-	0.0899	-	0.0826	-	-	-0.0656	0.0565	-0.2194	-0.1282	-	0.0575	-0.1144	0.0552	-0.1356	-	0.0956	0.0723	-
Brachyspira murdochii DSM 12563 uid48819	3241804	27.75	NC_014150	I	W	NC_012225	3000694	27.06	0.1938	1857	-	-0.0575	0.0823	0.1365	0.0640	-	-	-0.0632	0.0611	-0.0648	-	-	0.0852	-0.1378	-	-0.0901	-	-	-	-
Cellulomonas flavigena DSM 20109 uid48821	4123179	74.29	NC_014151	A	C	NC_015514	4266344	74.72	0.2148	899	-0.0964	-0.0652	-0.1889	-0.1200	-	-	-0.0809	0.1048	-	-	-0.0864	-0.0967	-	-	-	-	-	-	0.1047	-

Arcobacter nitrofigilis DSM 7299 uid49001	3192235	28.36	NC_014166	K	W	NC_009850	2341251	27.05	0.2670	1188	0.1365	-0.1043	-	-	0.0597	0.1117	-	-0.1469	0.0622	-0.2050	-0.1673	-	-	-0.1244	-	-0.1147	-	-	-	-
Bifidobacterium longum JDM301 uid49131	2477838	59.81	NC_014169	A	C	NC_013714	2636367	58.54	0.2341	870	-0.1520	-0.0735	-	-0.0861	-	-	-	0.1692	-0.1182	-	-	-0.1917	-0.1256	-	0.0667	-	0.0898	-	0.1378	-
Desulfurivibrio alkaliphilus AHT2 uid49487	3097763	60.29	NC_014216	L	W	NC_006138	3523383	46.81	0.2599	693	-0.0834	-	-0.0805	-0.1166	0.2696	-	-	0.1278	-	-	-	-	-0.1392	-	0.0788	-	-	-	0.0992	-
Arcanobacterium haemolyticum DSM 20595 uid49489	1986154	53.13	NC_014218	A	C	NC_014246	2146480	55.41	0.2841	452	-0.2403	-	0.1262	-0.1419	0.1244	-0.0891	-	0.1563	-	-	-0.1680	-	-0.1602	-	-	-	0.0953	0.1317	-	-
Bacillus selenitireducens MLS10 uid49513	3592487	48.67	NC_014219	L	C	NC_016023	3552226	46.49	0.1457	922	-0.1414	-	-	-	0.1720	-	-	0.0903	-	-0.0654	-	-	-	-0.0903	-	-	-	0.1058	0.1503	-
Croceibacter atlanticus HTCC2559 uid49661	2952962	33.90	NC_014230	L	C	NC_009613	2861988	32.54	0.2824	1175	-	-0.1984	0.0675	-	0.1171	-	-	-0.0762	0.1203	-0.2167	-0.1053	-0.0799	-	-0.1029	0.0606	-0.1398	-	-	-	0.0630
Acinetobacter DR1 uid50119	4152543	38.73	NC_014259	L	C	NC_010410	3936291	39.38	0.0808	2376	0.0928	-	-	-	-	-	0.0477	-	-	-0.1617	-0.0817	-0.0557	-	-0.0741	0.0779	-0.0722	-	-	-	-
Dehalogenimonas lykanthroporepellens BL DC 9 uid48131	1686510	55.04	NC_014314	A	W	NC_015682	1634377	30.59	0.1694	311	-	-	-	-0.1095	0.2463	-	-	-	-	-	-	-	-0.1334	0.1262	-	-	-	-	-	-
Amycolatopsis mediterranei U32 uid50565	10236715	71.29	NC_014318	A	C	NC_009142	8212805	71.15	0.1798	1281	-0.0644	-0.0541	-0.0932	-0.1182	-	-	-	0.1828	-	-	-0.1295	-0.0686	-0.0618	-	-	-	-	-	0.0980	-
Clostridium ljungdahlii DSM 13528 uid50583	4630065	31.11	NC_014328	K	W	NC_015977	3592125	48.50	0.1952	702	-	-	0.1959	0.1312	0.0864	0.1094	0.1096	-0.0962	-	-0.1023	-0.1129	-	-	-	-	-0.1444	-	0.1224	0.1021	-
Corynebacterium pseudotuberculosis FRC41 uid50585	2337913	52.19	NC_014329	A	C	NC_012704	2446804	57.46	0.3498	636	-0.1016	-	0.0711	-	-	-	0.1098	0.1647	-	-0.1539	-0.1304	-0.1340	-0.1625	-	0.1587	-	-	0.1305	0.2015	0.0996
Brachyspira pilosicoli 95 1000 uid50609	2586443	27.90	NC_014330	I	W	NC_012225	3000694	27.06	0.2602	1533	-	-0.1138	0.0974	0.0686	-	0.0488	-	-0.1281	0.0523	-0.1477	-	-	0.1137	-0.1358	-	-0.1318	-	-	-	-0.0583
Candidatus Nitrospira defluvii uid51175	4317083	59.03	NC_014355	L	W	NC_011296	2003803	34.13	0.1876	474	-0.1733	-	-0.1971	-	0.1483	-	-	-	-	-	-	-	-0.1215	0.1264	-	-0.0892	-	0.1546	-	-
Desulfarculus baarsii DSM 2075 uid51371	3655731	65.70	NC_014365	A	W	NC_011768	6517073	54.48	0.2476	950	-	-	-0.1344	-0.1123	0.2577	-	-	-	-0.0638	-	-	-0.0908	-0.1390	-	-	-	-	0.0822	0.1492	-
Brevundimonas subvibrioides ATCC 15264 uid42117	3445263	68.42	NC_014375	A	C	NC_011144	3996255	71.35	0.2350	1095	-0.1213	-0.1258	-0.1220	-0.0999	-	-	-	0.2120	-	-	-0.0827	-0.0632	-0.0905	0.0627	-	-	-	-	0.1315	-
Clostridium saccharolyticum WM1 uid51419	4662871	45.00	NC_014376	L	W	NC_010001	4847594	35.35	0.1518	1315	-0.1002	-0.0888	-	-	0.1386	0.0556	0.1023	-	0.0637	-0.0741	-0.1134	-	-	-	0.0622	-0.1703	-	-	0.0669	-
Caldicellulosiruptor obsidiansis OB47 uid51501	2532343	35.24	NC_014392	I	W	NC_014657	2428903	35.44	0.2323	1261	0.1083	-	-	-	-	-0.1053	-	-0.1009	0.1757	-0.0975	-0.0774	-	0.0698	-0.0947	0.1077	-0.0635	-	0.0710	-	-0.1359
Clostridium cellulovorans 743B uid51503	5262222	31.21	NC_014393	I	W	NC_014376	4662871	45.00	0.2088	904	-	-0.1055	0.1288	-	0.1266	-	0.0758	-0.0994	-	-0.1276	-	-	0.0815	-0.1276	0.0705	-0.2069	-	0.0727	-	-
Bacillus subtilis spizizenii W23 uid51879	4027676	43.89	NC_014479	L	C	NC_014639	4168266	43.22	0.0979	2393	-	0.0415	-	-0.0608	-	0.0830	0.0678	-	-	-0.1807	-0.0556	-0.0555	-	-0.0430	0.0792	-0.0954	0.0780	-	0.0964	-
Dickeya dadantii 3937 uid52537	4922802	56.30	NC_014500	L	C	NC_012912	4813854	54.52	0.0932	2708	0.0644	0.0397	-0.0472	-	-	-0.0445	-	0.0796	-	-0.1050	-	-0.0704	-0.0402	0.0786	0.1404	0.0632	-	-0.1067	0.1066	0.0534
Bacillus amyloliquefaciens DSM 7 uid53535	3980199	46.08	NC_014551	L	C	NC_014639	4168266	43.22	0.1067	2155	-	-	-0.0602	-0.0738	-	-	0.0961	0.1076	-	-0.1308	-	-0.0943	-	-	0.0873	-0.0530	0.0814	-0.0650	0.1045	-
Clostridium sticklandii DSM 519 uid59585	2715461	33.30	NC_014614	I	W	NC_008555	2814130	36.35	0.2113	531	-	-	0.1476	-	0.1350	0.1173	0.1013	-0.1030	-	-0.1145	-	-	-	-0.2222	-	-0.1807	-	0.1985	-	-
Bifidobacterium bifidum S17 uid59545	2186882	62.76	NC_014616	A	C	NC_008618	2089645	59.18	0.2363	754	-0.1025	-0.1312	-0.0789	-	0.1285	-	0.0797	0.1287	-	0.0947	-	-0.1105	-	0.1084	0.1728	-	-	-	0.1293	-
Enterobacter cloacae SCF1 uid59969	4814049	57.02	NC_014618	L	C	NC_015663	5280350	54.85	0.0958	2835	-0.0432	-	-0.0599	-	-	-0.0885	-	0.0915	-0.0759	-0.1161	-0.1171	-0.0875	-0.0633	-	0.0944	-	-	-	0.0904	-
Bifidobacterium bifidum PRL2010 uid59883	2214656	62.67	NC_014638	A	C	NC_008618	2089645	59.18	0.2968	738	-0.1313	-0.1630	-0.1454	-	0.1235	-	0.0769	0.1151	-0.0801	-	-	-0.1762	-0.1138	-	0.1126	-	-	-	0.1147	-
Bacillus atrophaeus 1942 uid59887	4168266	43.22	NC_014639	L	C	NC_014551	3980199	46.08	0.1050	2155	-	-	-	-	0.0694	0.0448	0.0835	-	-	-0.1369	-	-0.1178	-	-0.0466	0.1233	-0.0503	0.0807	-0.0848	0.0982	-
Caldicellulosiruptor hydrothermalis 108 uid60157	2770676	36.11	NC_014652	I	W	NC_009437	2970275	35.25	0.2120	1371	0.0810	-0.0619	-	0.0674	0.0950	-	0.0914	-0.1090	0.1438	-0.1136	-	-	-	-0.0686	0.0973	-0.0948	-	-	0.0873	-0.0866
Bifidobacterium longum BBMN68 uid60163	2265943	59.95	NC_014656	A	C	NC_014638	2214656	62.67	0.2267	855	-0.0883	-	-0.1917	-	0.1031	-	0.1213	0.2046	-0.0917	-	-0.1010	-0.1565	-	-	0.1590	-	-	-	-	-
Caldicellulosiruptor owensensis OL uid60165	2428903	35.44	NC_014657	I	W	NC_014392	2532343	35.24	0.2394	1261	0.1230	-	-	0.0586	-	-0.1101	-	-0.1267	0.1774	-0.0858	-0.1014	-	-	-0.1164	0.0910	-0.0677	-	0.0615	-	-0.1197
Caldicellulosiruptor kronotskyensis 2002 uid60491	2843785	35.10	NC_014720	I	W	NC_009437	2970275	35.25	0.2228	1387	0.0810	-	0.0958	0.1068	0.1320	-	0.0573	-0.1055	0.1074	-0.0753	-	-	-	-	0.0815	-0.1172	-	-	0.1574	-0.0959
Bacillus cellulosilyticus DSM 2522 uid43329	4681672	36.52	NC_014829	L	C	NC_000964	4215606	43.51	0.2439	1155	-0.0840	-	0.1506	-	0.0845	-	-	-	0.1188	-0.1397	-0.1438	-	-	-0.1128	0.0690	-0.1094	-	0.0935	0.0875	-
Desulfovibrio aespoeensis Aspo 2 uid42613	3629109	62.56	NC_014844	L	W	NC_011769	4040304	67.11	0.2210	1214	-	-0.0705	-0.1589	-0.1140	0.1912	-	-	0.1320	-0.0694	-0.0930	-	-	-0.0751	-	-	-	0.0715	0.0695	0.1289	-
Candidatus Blochmannia vafer BVAF uid62083	722593	27.51	NC_014909	I	W	NC_011833	642122	26.29	0.4302	310	-	-	-	-	0.2714	-	-	-0.2579	-	-0.2488	-0.1741	-0.1007	-	-0.2159	-	-	-	0.3033	-	-

Bartonella clarridgeiae 73 uid62131	1522743	35.73	NC_014932	L	W	NC_005955	1581384	38.80	0.3281	824	0.1466	-	-	-	0.0820	-	-	-0.2007	-	-0.2721	-0.1986	-	-	-0.1868	0.1278	-0.1199	0.0882	0.0657	-	-
Bacteroides helcogenes P 36 108 uid62135	3998906	44.72	NC_014933	L	W	NC_015164	4242803	46.65	0.1401	1467	-	-0.0829	-	-	0.1030	-	0.0720	-	-	-0.1151	-0.0861	-	0.1079	-	0.1395	-0.1011	-	0.1230	0.0901	0.0549
Cellulophaga algicola DSM 14237 uid62159	4888353	33.77	NC_014934	L	C	NC_014041	5128187	36.22	0.2431	1607	0.0846	-0.0573	0.0627	0.0707	0.1206	-	-	-	0.1051	-0.1789	-0.1237	-0.0654	0.0631	-0.1656	-	-0.1253	-	-	0.0715	0.0701
Deinococcus maricopensis DSM 21211 uid62225	3498530	69.83	NC_014958	A	C	NC_012526	2819842	63.39	0.2472	1156	-0.1239	-0.0571	-0.1916	-	0.1381	-	-	0.0870	-	-	-0.0964	-0.1171	-0.0947	0.0755	-	-	0.0933	-	0.1048	0.0688
Anaerolinea thermophila UNI 1 uid62245	3532378	53.85	NC_014960	L	C	NC_011026	3293456	45.04	0.1737	399	-	-	0.1070	-	0.1814	-	0.1177	-	-	-	-0.1168	-	-0.2138	-	-	-0.1415	-	0.1465	0.1593	-
Desulfobulbus propionicus DSM 2032 uid62265	3851869	58.93	NC_014972	L	W	NC_009943	3944167	56.17	0.2050	768	-	-	-	-	0.2782	-	-	0.1713	-	-0.1312	-0.1007	-	-0.0770	-	-	-	0.0820	0.1477	-	-
Bacillus subtilis BSn5 uid62463	4093599	43.85	NC_014976	L	C	NC_002570	4202352	43.69	0.2237	1295	-	-	0.1115	-0.0801	0.1247	-	-	-	0.0800	-0.1083	-0.1146	-0.1192	-	-0.1607	0.0530	-	0.0852	-	0.0700	0.0681
Bifidobacterium longum JCM 1217 uid62695	2385164	60.33	NC_015067	A	C	NC_014638	2214656	62.67	0.1373	860	-	-	-0.1738	-	-	-	-	0.1712	-	-	-	-0.1730	-	-	0.1075	-	-	-	-	-
Acidovorax avenae ATCC 19860 uid42497	5482170	68.82	NC_015138	A	C	NC_008752	5352772	68.53	0.1992	3054	-	-	-0.0885	-0.0435	-0.0674	-0.0355	0.0809	0.1411	-0.0481	-	-	-0.0462	-0.0554	0.0442	0.0916	0.1692	-	-0.0712	0.0984	-0.0451
Cellulophaga lytica DSM 7489 uid63401	3765936	32.11	NC_015167	L	C	NC_008571	3798465	36.61	0.2495	1499	0.0791	-0.1113	-	0.1289	0.0805	-	-	-0.0820	0.1274	-0.1626	-0.1141	-0.0687	0.0912	-0.1484	-	-0.1051	-	-	-	-
Desulfurobacterium thermolithotrophum DSM 11699 uid63405	1541968	34.95	NC_015185	K	W	NC_014926	1682965	52.11	0.3510	781	0.1629	-	-	0.0956	0.0877	-0.0928	-	-0.1592	0.1363	-	-0.0738	-	-	-0.1331	0.1883	-0.1097	-	-	-	-
Clostridium lentocellum DSM 5427 uid49117	4714237	34.32	NC_015275	L	W	NC_015977	3592125	48.50	0.1590	964	-	-	0.1782	-	-	-	0.0707	-0.1031	-	-0.1320	-0.1176	-	0.0876	-0.1387	-	-0.1306	-	0.1390	-	-
Aerococcus urinae ACS 120 V Col10a uid64757	2080974	41.88	NC_015278	L	W	NC_009513	1999618	38.87	0.2783	459	-	-	0.0895	0.0845	0.1353	0.1126	-	-	-	-0.1425	-0.1519	-0.2040	-	-0.1962	-	-0.1449	-	0.1364	0.1319	-
Candidatus Pelagibacter IMCC9063 uid66305	1284727	31.67	NC_015380	K	W	NC_012416	1445873	35.16	0.2689	292	0.1967	-	-	-	0.1989	-	-	-	0.1355	-0.1615	-	-	-0.1129	-0.2663	-	-	-	-	-	-
Desulfobacca acetoxidans DSM 11109 uid65785	3282536	52.89	NC_015388	L	W	NC_007759	3179300	51.46	0.1772	534	-	-	-	-0.1227	0.2148	-	-	-	-	-	-0.1245	-	-0.1008	-	-	-	0.0970	0.1806	0.1200	-
Coriobacterium glomerans PW2 uid65787	2115681	60.39	NC_015389	A	W	NC_014363	2051896	64.70	0.1174	562	-0.1158	-0.0824	-	-0.1372	-	-	0.1017	-	-	-	-	-	-	-	0.1576	-	-	-	-	-
Chlamydophila pecorum E58 uid66295	1106197	41.08	NC_015408	L	W	NC_002620	1072950	40.34	0.3051	546	-	-	-	-0.0790	0.1744	-	0.1051	-	-	-0.1850	-0.1476	-	-	-0.1239	0.2379	-	0.0999	-	0.0814	-
Aeromonas veronii B565 uid66323	4551783	58.72	NC_015424	L	C	NC_009348	4702402	58.51	0.1309	2561	-	-	-0.1367	-	-	-0.0466	0.0417	0.0498	-0.0700	-0.0799	-0.0762	-0.0589	-0.0752	-	-	-	0.1014	-	0.1406	-
Cellulomonas fimi ATCC 484 uid66779	4266344	74.72	NC_015514	A	C	NC_014151	4123179	74.29	0.2121	899	-0.0925	-	-0.1748	-0.1129	-	-	-0.0706	0.1263	-	-	-0.0984	-	-	-	-	-	-	-	0.0794	-
Alteromonas SN2 uid67349	4972148	43.51	NC_015554	L	C	NC_011138	4448980	44.89	0.1545	2037	0.0491	-	-	-	-	-0.0605	-	-	-	-0.2057	-0.1655	-0.0757	-	-0.1269	0.1493	-	-	0.0732	-	0.0908
Delftia Cs1 4 uid67319	6685842	66.72	NC_015563	A	C	NC_010002	6767514	66.48	0.0805	3428	-0.0517	-	-	-	-	-	0.0720	0.0631	-	-	-	-0.0442	-0.0705	-	0.0628	0.1073	0.0583	-0.0604	0.0882	-
Desulfotomaculum carboxydivorans CO 1 SRB uid67317	2892255	46.63	NC_015565	L	W	NC_015573	3601386	54.88	0.1602	661	-	-	-	-	0.2772	-	-	-	-	-	-	-0.1192	-	-0.1145	-	-	0.1047	0.1138	-	-
Desulfotomaculum kuznetsovii DSM 6115 uid67357	3601386	54.88	NC_015573	L	W	NC_015565	2892255	46.63	0.1701	661	-	-0.0995	-0.1199	-	0.2802	-0.0940	-	-	-	-	-	-	-	-	-	-	-	0.1703	0.1315	-
Erysipelothrix rhusiopathiae Fujisawa uid68021	1787941	36.56	NC_015601	L	W	NC_013504	1755993	34.49	0.2546	373	-	-	0.1372	-	-	0.1433	-	-	-	-0.1446	-0.2458	-0.1625	0.1517	-0.1546	-	-0.1486	-	0.2012	-	-
Bacillus coagulans 2 6 uid68053	3073079	47.29	NC_015634	L	C	NC_016047	4207222	43.82	0.1253	1153	-	-0.0634	-	-	0.1459	-	-	0.0832	-	-0.1205	-0.1176	-0.0765	-	-0.1282	-	-	0.0648	-	-	-
Buchnera aphidicola Cinara tujafilina uid68101	444925	23.03	NC_015662	I	W	NC_015735	538294	43.52	0.5206	185	0.1572	-	0.2415	-	0.1992	-	-	-	-	-0.1613	-	-0.1807	-	-0.2154	-	-0.1774	-	-	-	-
Enterobacter aerogenes KCTC 2190 uid68103	5280350	54.85	NC_015663	L	C	NC_014618	4814049	57.02	0.0983	2835	-	-	-	0.0602	-	-0.0543	0.0467	0.1102	-	-0.1141	-0.0737	-0.0967	-0.0694	-	0.0548	-	0.0884	-	0.0959	0.0439
Corynebacterium resistens DSM 45100 uid50555	2601311	57.09	NC_015673	A	C	NC_007164	2462499	61.40	0.1897	821	-	-	-	-	-	-0.0900	-	0.1915	-	-	-0.1370	-0.0935	-0.1111	-	0.1997	-	-	-	0.0816	-
Corynebacterium ulcerans BR AD22 uid68291	2606374	53.40	NC_015683	A	C	NC_007164	2462499	61.40	0.2341	698	-	-	-	-	0.0943	-0.1554	-	0.1570	-	-0.0869	-	-0.1413	-0.1464	-	0.1445	-	-	0.1251	0.1058	-
Candidatus Midichloria mitochondrii IricVA uid68687	1183732	36.55	NC_015722	L	W	NC_009465	1022154	31.59	0.2564	239	-	0.1350	-	0.1605	-	-	-	-	-	-0.2832	-0.1423	-0.1566	-	-	-	-0.2126	-	0.2098	0.1358	-
Candidatus Moranella endobia PCIT uid68739	538294	43.52	NC_015735	L	W	NC_014109	574390	28.48	0.1399	195	-	-	-	-	0.1977	-	-	-	-	-0.1993	-	-0.1953	-	-0.1710	-	-	-	0.1785	-	-
Clostridium SY8519 uid68705	2835737	50.74	NC_015737	A	W	NC_004557	2799251	28.75	0.1801	431	-0.1999	-	-	-0.1347	0.1453	-	0.1826	-	-	-	-	-	0.1147	-	0.1100	-0.1278	-	0.1619	-	-
Eggerthella YY7918 uid68707	3123671	56.20	NC_015738	A	W	NC_013204	3632260	64.20	0.1622	1238	-0.1035	-	-0.1296	-	-	-	0.0755	-	-	-0.1073	-0.1458	-	-0.0651	-	0.2311	-0.0696	-	0.0603	-	-

Chlamydia trachomatis L2c uid68843	1038313	41.33	NC_015744	L	W	NC_015408	1106197	41.08	0.3001	569	-	-	-0.1554	-	0.1247	-	0.1558	-	-	-0.1599	-0.1541	-	-0.1123	-0.1119	0.2299	-	-	-	0.0960	-
Capnocytophaga canimorsus Cc5 uid70727	2571406	36.11	NC_015846	L	C	NC_013162	2612925	39.59	0.2238	1084	-	-0.1127	-	-	0.1305	-	-	-	0.1482	-0.2126	-0.1299	-0.0839	-	-0.1225	-	-0.0958	-	0.0755	0.0741	-
Collimonas fungivorans Ter331 uid70793	5186898	59.56	NC_015856	A	C	NC_009138	3424307	54.29	0.2320	1206	-0.1310	-0.0792	-0.1563	-	0.0727	-	-	0.2227	-0.0991	-	-0.0804	-	-	-	0.1024	0.0571	-	-	0.1235	0.0620
Corynebacterium variabile DSM 44702 uid62003	3433007	67.15	NC_015859	A	C	NC_003450	3309401	53.81	0.2720	733	-0.1462	-	-	-0.0771	-	-0.0841	-	0.1672	-0.0724	-	-	-0.0954	-0.2057	-	-	-	-	-	0.1273	-
Candidatus Arthromitus SFB mouse Japan uid71379	1620005	28.26	NC_015913	I	W	NC_011295	1424912	44.77	0.1858	173	-	-	-	-	-	-	-	-	-	-	-	-0.1698	-	-	-	-	-	-	-	-
Acidithiobacillus ferrivorans SS3 uid67387	3207552	56.59	NC_015942	A	C	NC_011761	2982397	58.77	0.1469	1430	-	0.0526	-0.0858	-	-	-	-	0.1325	-0.0777	-0.1215	-0.0737	-0.0626	-0.0679	-	0.1126	0.1213	-	-	0.1830	0.0645
Burkholderia JV3 uid72473	4544477	66.89	NC_015947	A	C	NC_003919	5175554	64.77	0.3275	1762	-0.1362	-0.0710	-0.1636	-0.0820	0.0941	-	-	0.1211	-0.1067	-	-0.0604	-0.0909	-0.0899	-	0.0514	0.0492	0.0929	0.0625	0.1842	-0.0479
Caldicellulosiruptor lactoaceticus 6A uid60575	2674809	36.13	NC_015949	L	W	NC_014392	2532343	35.24	0.1764	1208	0.1129	-	-	0.0784	-	-0.0821	-	-0.0602	0.1735	-	-	-	0.0798	-0.0887	0.0960	-0.1770	-	-	0.0665	-
Candidatus Arthromitus SFB rat Yit uid73425	1515556	27.98	NC_016012	I	W	NC_015759	1422478	35.48	0.2265	254	-	-0.1212	0.2019	-	-	-	-	-	-	-0.2269	-0.2150	-	-	-0.1320	-	-0.1735	-	0.1976	-	-
Bacillus coagulans 36D1 uid54335	3552226	46.49	NC_016023	L	W	NC_014219	3592487	48.67	0.1398	922	-	-	-	-	0.1535	-	-	0.0760	-	-0.0957	-0.1378	-0.0805	-	-0.1903	-	-	-	-	0.0639	-
Bacillus subtilis spizizenii TU B 10 uid73967	4207222	43.82	NC_016047	L	C	NC_015634	3073079	47.29	0.1616	1153	-	-	0.0630	-0.0810	0.1081	-	-	-	-	-0.1841	-	-0.0726	-	-0.2092	0.1060	-	0.0616	-	-	-
Acidaminococcus intestini RyC MR95 uid74445	2487765	50.02	NC_016077	L	W	NC_013740	2329769	55.84	0.1168	1017	-	-	-0.1398	-0.1679	0.1223	-	-	0.0968	-	-0.1030	-0.0708	-	-	-	-	-	-	0.0674	0.1096	-
Escherichia coli str. K-12 substr. MG1655	4639675	50.80	NC_000913	L	C	NC_014479	4027676	43.89	0.1827	610	-	-0.0826	-	-	0.2288	-	-	-	-	-0.1066	-	-	-0.1110	-0.0928	-	-	-	0.2024	0.1036	-
Mycobacterium tuberculosis H37Rv	4411532	65.60	NC_000962	A	C	NC_015125	3982034	70.28	0.2968	351	-0.2747	-	-0.1307	-	-	-	-	0.2024	-	-	-0.2111	-	-	-	-	-	-	0.1552	-	-
Neisseria meningitidis MC58	2272360	51.50	NC_003112	A	C	NC_012115	1676444	33.51	0.0524	148	-	-	-	-	-	-	-	-	-	-	-	-	-	-	-	-	-	-	-	-
Streptococcus pyogenes M1 GAS	1852441	38.50	NC_002737	L	C	NC_007350	2516575	33.24	0.2582	455	-	-	-	-0.0883	0.1123	0.0940	-	-	0.1184	-0.1827	-0.1462	-0.1428	-	-0.2169	-	-0.2209	-	0.2169	-	-
Desulfovibrio vulgaris str. Hildenborough	3570858	63.10	NC_002937	A	W	NC_006832	1512977	27.48	0.2547	127	-	-0.2295	-0.2837	-0.2112	-	-	-	-	-	-	-	-	-0.2455	-	-	-	-	0.2147	-	-

Table S2 Total decision coefficients and amino acid contributions for the linear models between evolutionary rates and multi amino acid compositions specially for *E.coli* strains

The details for twenty-nine *E.coli* strains were listed in the following table. Their linear models are constructed based on two different orthologous chromosomes - NC_014479 and NC_011740.

Organism	GC_content	Chromosome	Richest AA	Rarest AA	Orthologous Chromosome	R^2	Orthologous gene numbers	The contributions of 20 amino acids to the linear models																			
								K	H	I	N	L	M	C	A	F	G	D	E	Y	R	S	P	Q	V	W	T
Escherichia coli O157:H7 str. EDL933	50.51	NC_002655	L	C	NC_014479	0.1813	596	-	-0.10	-	-	0.22	-0.09	-	-	-	-0.11	-	-	-0.12	-0.09	-	-	-0.13	0.22	0.09	-
Escherichia coli O157 H7 Sakai uid57781	50.65	NC_002695	L	C	NC_014479	0.1718	606	-	-	-	-	0.22	-0.11	-	-	-	-0.12	-	-	-0.13	-0.10	-	-	-0.14	0.16	-	-
Escherichia coli CFT073 uid57915	50.43	NC_004431	L	C	NC_014479	0.1614	534	-0.11	-	-	-	0.24	-0.11	-	-	-		-	-	-0.12	-0.12	-	-	-0.15	0.19	-	-
Escherichia coli UTI89 uid58541	50.50	NC_007946	L	C	NC_014479	0.1808	536	-	-	-	-	0.23	-	-	-	-	-0.10	-	-	-0.16	-0.09	-	-	-0.11	0.23	0.10	-
Escherichia coli 536 uid58531	50.35	NC_008253	L	C	NC_014479	0.1965	608	-	-0.10	-	-	0.26	-	0.08	-	-		-	-	-0.13	-	0.08	-	-0.10	0.19	0.09	-
Escherichia coli APEC O1 uid58623	50.51	NC_008563	L	C	NC_014479	0.1467	552	-	-	-	-	0.22	-		-	-		-	-	-0.14	-	-	-	-0.11	0.16	-	-
Escherichia coli HS uid58393	50.71	NC_009800	L	C	NC_014479	0.2291	599	-0.12	-0.10	-	-0.08	0.22	-0.15	0.08	-	-	-0.13	-	-	-0.15	-0.09	-	-	-0.14	0.21	0.11	-
Escherichia coli E24377A uid58395	50.51	NC_009801	L	C	NC_014479	0.2027	602	-	-0.09	-	-0.08	0.21	-0.09	0.08	-	-	-0.10	-	-	-0.15	-0.12	-	-		0.24	0.15	-
Escherichia coli ATCC 8739 uid58783	50.97	NC_010468	L	C	NC_014479	0.2003	604	-	-0.10	-	-	0.23	-0.11	0.08	-	-	-0.14	-	-	-0.10	-0.10	-	-	-0.12	0.20	0.09	-
Escherichia coli K 12 substr DH10B uid58979	50.74	NC_010473	L	C	NC_014479	0.1654	593	-	-0.10	-	-	0.24			-	-	-0.11	-	-	-0.10	-0.09	-	-	-	0.20	0.09	-
Escherichia coli SMS 3 5 uid58919	50.42	NC_010498	L	C	NC_014479	0.2063	613	-	-0.09	-	-	0.30	-0.09		-	-	-0.11	-	-	-0.08	-	-	-	-0.11	0.21	0.09	-
Escherichia coli O157 H7 EC4115 uid59091	50.42	NC_011353	L	C	NC_014479	0.1930	599	-0.09	-0.09	-	-	0.22	-0.10		-	-	-0.13	-	-	-0.13	-0.10	-	-	-0.13	0.21	0.09	-
Escherichia coli SE11 uid59425	50.63	NC_011415	L	C	NC_014479	0.2291	607	-0.10	-0.11	-	-	0.25	-0.12	0.09	-	-	-0.16	-	-	-0.14	-0.11	-	-	-0.13	0.24	0.10	-
Escherichia coli O127 H6 E2348 69 uid59343	50.61	NC_011601	L	C	NC_014479	0.1914	606	-	-0.09	-	-	0.23	-0.10		-	-	-0.11	-	-	-0.12	-0.11	-	-	-0.12	0.18	0.10	-

Escherichia coli IAI1 uid59377	50.66	NC_011741	L	C	NC_014479	0.2256	608	-	-0.08	-	-	0.27	-0.11	0.09	-	-	-0.14	-	-	-0.12	-0.10	-	-	-0.09	0.23	-	-
Escherichia coli S88 uid62979	50.66	NC_011742	L	C	NC_014479	0.1700	605	-	-0.09	-	-	0.22	-	0.08	-	-	-0.09	-	-	-0.15	-0.12	-	-	-0.11	0.17	0.09	-
Escherichia coli ED1a uid59379	50.48	NC_011745	L	C	NC_014479	0.1671	600	-	-	-	-	0.24	-		-	-		-	-	-0.12	-0.10	-	-	-	0.16	-	-
Escherichia coli 55989 uid59383	50.56	NC_011748	L	C	NC_014479	0.1856	601	-	-0.10	-	-	0.24	-	0.09	-	-	-0.10	-	-	-0.11	-0.08	-	-	-	0.23	0.11	-
Escherichia coli IAI39 uid59381	50.38	NC_011750	L	C	NC_014479	0.1992	606	-	-	-	-	0.27	-		-	-		-	-	-0.09	-	-	-	-0.11	0.21	-	-
Escherichia coli UMN026 uid62981	50.67	NC_011751	L	C	NC_014479	0.1644	606	-	-	-	-	0.25	-	0.09	-	-	-0.10	-	-	-0.08	-	-	-	-0.11	0.16	-	-
Escherichia coli BW2952 uid59391	50.74	NC_012759	L	C	NC_014479	0.1734	602	-	-0.11	-	-	0.25	-	0.08	-	-	-0.09	-	-	-0.12	-0.09	-	-	-	0.21	0.10	-
Escherichia coli BL21 Gold DE3 pLysS AG uid59245	50.88	NC_012947	L	C	NC_014479	0.1678	597	-	-0.11	-	-	0.21	-	0.08	-	-	-0.09	-	-	-0.11	-	-	-	-	0.20	0.10	-
Escherichia coli B REL606 uid58803	50.70	NC_012967	L	C	NC_014479	0.1900	603	-	-0.13	-	-0.08	0.20	-0.09	-	-	-	-0.12	-	-	-0.11	-0.10	-	-	-	0.18	0.10	-
Escherichia coli O157 H7 TW14359 uid59235	50.45	NC_013008	L	C	NC_014479	0.1898	606	-	-0.11	-	-	0.20	-0.11	-	-	-	-	-0.10	-	-0.13	-0.09	-0.09	-	-0.14	0.18	0.09	-
Escherichia coli O103 H2 12009 uid41013	50.46	NC_013353	L	C	NC_014479	0.1899	606	-	-	-	-	0.30	-	0.08	-	-	-	-	-	-0.12	-0.11	-	-	-	0.21	0.12	-
Escherichia coli O26 H11 11368 uid41021	50.69	NC_013361	L	C	NC_014479	0.2217	612	-	-0.10	-	-	0.28	-0.08	0.09	-	-	-0.12	-	-	-0.14	-0.10	-	-	-	0.20	0.12	-
Escherichia coli O111 H 11128 uid41023	50.55	NC_013364	L	C	NC_014479	0.2026	609	-	-	-	-	0.28	-0.09	0.09	-	-	-0.12	-	-	-0.14	-0.10	-	-	-	0.21	0.11	-
Escherichia coli O55 H7 CB9615 uid46655	50.53	NC_013941	L	C	NC_014479	0.1803	589	-	-0.09	-	-	0.22	-	-	-	-	-0.09	-	-	-0.11	-	-	-	-0.11	0.21	0.10	-
Escherichia coli str. K-12 substr. MG1655	50.80	NC_000913	L	C	NC_014479	0.1827	610	-	-0.08	-	-	0.23	-	-	-	-	-0.11	-	-	-0.11	-0.09	-	-	-	0.20	0.10	-
Escherichia coli O157:H7 str. EDL933	50.51	NC_002655	L	C	NC_011740	0.0604	2219	-	-	-	-	-	-	0.10	-	-	-0.12	-	-0.08	-	-	0.11	-	0.09	-	0.05	0.07
Escherichia coli O157 H7 Sakai uid57781	50.65	NC_002695	L	C	NC_011740	0.0619	2286	-	-	-	-	-	-	0.10	-	-	-0.11	-	-0.10	-	0.05	0.11	-	0.07	-	0.08	0.07
Escherichia coli CFT073 uid57915	50.43	NC_004431	L	C	NC_011740	0.0549	2056	-	0.07	-	-0.04	-	-	0.08	-	-0.06	-0.10	-	-0.08	-	-	0.12	-	-	-0.08	-	-
Escherichia coli UTI89 uid58541	50.50	NC_007946	L	C	NC_011740	0.0414	2091	-	-	-	-	-	-	0.10	-	-	-0.07	-0.04	-0.07	-	-	0.10	-0.05	-	-0.09	-	-
Escherichia coli 536 uid58531	50.35	NC_008253	L	C	NC_011740	0.0689	2312	-	0.05	-	-	-	-0.07	0.09	-	-	-0.12	-0.04	-0.10	-	-	0.10	-	0.09	-	0.06	0.04
Escherichia coli APEC O1 uid58623	50.51	NC_008563	L	C	NC_011740	0.0706	2161	-	0.05	0.06	-	-	-0.07	0.09	-	-	-0.07	-	-	-	-	0.12	-	0.07	-	0.14	0.13
Escherichia coli HS uid58393	50.71	NC_009800	L	C	NC_011740	0.0564	2244	-	-	-	-	-	-0.07	0.11	-	-	-0.13	-	-0.09	-	-	0.09	-	0.08	-	0.05	-
Escherichia coli E24377A uid58395	50.51	NC_009801	L	C	NC_011740	0.0452	2307	-	-	-	-	-	-0.08	0.09	-	-	-0.11	-0.05	-0.08	-	-	0.07	-	-	-0.07	-	-
Escherichia coli ATCC 8739 uid58783	50.97	NC_010468	L	C	NC_011740	0.0592	2316	-	-	-	-	-	-0.06	0.08	-	-0.07	-0.12	-0.04	-0.07	-	-	0.08	-	0.07	-	0.11	0.05
Escherichia coli K 12 substr DH10B uid58979	50.74	NC_010473	L	C	NC_011740	0.0450	2258	-	-	-	-	-	-0.05	0.08	-	-	-0.12	-	-0.10	-	-	0.06	-	-	-0.06	0.05	-
Escherichia coli SMS 3 5 uid58919	50.42	NC_010498	L	C	NC_011740	0.0759	2293	-	-	-	-	-	-0.06	0.12	-	-0.06	-0.13	-0.05	-0.06	-	-	0.09	-	0.05	-	0.13	0.05
Escherichia coli O157 H7 EC4115 uid59091	50.42	NC_011353	L	C	NC_011740	0.0712	2239	-	0.05	-	-	-	-	0.10	-	-	-0.12	-	-0.07	-	-	0.11	-	0.10	-	0.11	0.04
Escherichia coli SE11 uid59425	50.63	NC_011415	L	C	NC_011740	0.0522	2302	-	-	-0.04	-	-	-0.05	0.08	-	-	-0.15	-0.06	-0.08	-	-	0.08	-	-	-	-	0.07
Escherichia coli O127 H6 E2348 69 uid59343	50.61	NC_011601	L	C	NC_011740	0.0416	2354	-	-	-	-	-	-	0.11	-	-	-0.07	-	-0.06	-	-	0.10	-	0.04	-	-	0.05
Escherichia coli IAI1 uid59377	50.66	NC_011741	L	C	NC_011740	0.0517	2329	0.05	-	-0.05	-	-	-0.05	0.10	-	-	-0.14	-0.08	-0.09	-	-	0.08	-	-	-	-	-
Escherichia coli S88 uid62979	50.66	NC_011742	L	C	NC_011740	0.0376	2390	-	-	-	-	-	-	0.09	-	-	-0.06	-	-0.06	-	0.05	0.11	-0.04	-	-0.05	-	-
Escherichia coli ED1a uid59379	50.48	NC_011745	L	C	NC_011740	0.0420	2335	-	0.05	-	-	-	-	0.10	-	-	-0.09	-	-0.06	-	-	0.10	-	0.05	-	-	0.06
Escherichia coli 55989 uid59383	50.56	NC_011748	L	C	NC_011740	0.0588	2312	-	0.04	-0.05	-	-	-	0.10	0.04	-	-0.15	-	-0.11	-	-	0.08	-	-	-	0.05	0.05

Escherichia coli IAI39 uid59381	50.38	NC_011750	L	C	NC_011740	0.0583	2292	-	-	-	-	-	-0.04	0.07	-	-	-0.12	-	-0.05	-	-	0.10	-	0.07	-	0.08	0.07
Escherichia coli UMN026 uid62981	50.67	NC_011751	L	C	NC_011740	0.0566	2374	0.07	-	-0.05	-	-	-0.04	0.09	-	-	-0.12	-0.06	-0.09	-	-	0.09	-	0.04	-	0.05	0.05
Escherichia coli BW2952 uid59391	50.74	NC_012759	L	C	NC_011740	0.0590	2311	0.06	-	-	-	-	-0.05	0.09	-	-	-0.12	-0.05	-0.09	-	-	0.08	-	0.08	-0.04	0.07	-
Escherichia coli BL21 Gold DE3 pLysS AG uid59245	50.88	NC_012947	L	C	NC_011740	0.0573	2293	-	-	-	-	-	-0.06	0.09	-	-	-0.12	-	-0.08	-	-	0.11	-	0.06	-	0.06	0.04
Escherichia coli B REL606 uid58803	50.70	NC_012967	L	C	NC_011740	0.0472	2257	-	-	-	-	-	-0.08	0.08	-	-	-0.10	-	-0.12	-	-	0.08	-	-	-0.06	-	-
Escherichia coli O157 H7 TW14359 uid59235	50.45	NC_013008	L	C	NC_011740	0.0787	2306	-	-	-	-	-	-	0.10	0.05	-	-0.14	-	-0.07	-	-	0.12	-	0.10	-	0.11	0.06
Escherichia coli O103 H2 12009 uid41013	50.46	NC_013353	L	C	NC_011740	0.0572	2377	0.05	-	-0.08	-	-	-0.06	0.08	-	-	-0.15	-0.06	-0.10	-	-	0.09	-	-	-	-	0.05
Escherichia coli O26 H11 11368 uid41021	50.69	NC_013361	L	C	NC_011740	0.0551	2364	0.05	-	-	-	-	-0.05	0.09	-	-	-0.13	-0.05	-0.09	-	0.06	0.10	0.04	-	-	0.05	0.06
Escherichia coli O111 H 11128 uid41023	50.55	NC_013364	L	C	NC_011740	0.0558	2351	-	-	-0.07	-	-	-	0.13	-	-	-0.14	-	-0.08	-	-	0.09	-	-	-	-	-
Escherichia coli O55 H7 CB9615 uid46655	50.53	NC_013941	L	C	NC_011740	0.0583	2234	-	0.04	-	-	-	-	0.09	-	-	-0.12	-	-0.07	-	-	0.11	-	0.07	-	0.08	0.07
Escherichia coli str. K-12 substr. MG1655	50.80	NC_000913	L	C	NC_011740	0.0630	2320	0.07	-	-	-	-	-0.07	0.10	-	-	-0.13	-0.07	-0.10	-	-	0.08	-	0.08	-0.05	0.07	-
